# The HY5-PIF Regulatory Module Coordinates Light and Temperature Control of Photosynthetic Gene Transcription

**DOI:** 10.1371/journal.pgen.1004416

**Published:** 2014-06-12

**Authors:** Gabriela Toledo-Ortiz, Henrik Johansson, Keun Pyo Lee, Jordi Bou-Torrent, Kelly Stewart, Gavin Steel, Manuel Rodríguez-Concepción, Karen J. Halliday

**Affiliations:** 1 Institute of Structural and Molecular Biology, SynthSys, University of Edinburgh, Edinburgh, United Kingdom; 2 Plant Physiology, Justus Liebig University, Senckernbergstr, Giessen, Germany; 3 Centre for Research in Agricultural Genomics (CRAG) CSIC-IRTA-UAB-UB, Campus UAB Bellaterra, Barcelona, Spain; Peking University, China

## Abstract

The ability to interpret daily and seasonal alterations in light and temperature signals is essential for plant survival. This is particularly important during seedling establishment when the phytochrome photoreceptors activate photosynthetic pigment production for photoautotrophic growth. Phytochromes accomplish this partly through the suppression of PHYTOCHROME INTERACTING FACTORS (PIFs), negative regulators of chlorophyll and carotenoid biosynthesis. While the bZIP transcription factor LONG HYPOCOTYL 5 (HY5), a potent PIF antagonist, promotes photosynthetic pigment accumulation in response to light. Here we demonstrate that by directly targeting a common promoter *cis*-element (G-box), HY5 and PIFs form a dynamic activation-suppression transcriptional module responsive to light and temperature cues. This antagonistic regulatory module provides a simple, direct mechanism through which environmental change can redirect transcriptional control of genes required for photosynthesis and photoprotection. In the regulation of photopigment biosynthesis genes, HY5 and PIFs do not operate alone, but with the circadian clock. However, sudden changes in light or temperature conditions can trigger changes in HY5 and PIFs abundance that adjust the expression of common target genes to optimise photosynthetic performance and growth.

## Author Summary

Plants, as sessile and photosynthetic organisms, have to constantly adjust their growth and development in response to the environment. While light and temperature are recognized as the most prominent environmental factors modulating plant photosynthetic metabolism, how the seasonal and daily adjustments are achieved is not understood. Global climate alterations will bring together the combination of light and temperature changes and will require an understanding of signal convergence. If we are to mitigate the impact of variable weather patterns on agriculture, it is critical to advance our understanding of the basis of plant responses to environmental variations. In our study we show that the antagonistic activity of key plant transcription factors involved in phytochrome red light photoreceptors signaling (PIFs and HY5) optimize photosynthetic pigment production in response to environmental cues. These light and temperature responsive transcription factors operate in cooperation with the circadian clock to regulate photosynthetic pigment production through a common gene promoter element.

## Introduction

Light and temperature are prominent cues that signify seasonal and climatic change, as well as the phase of the daily light/dark cycle. The ability to sense and integrate these external signals is essential for plant life cycle progression and ultimately, survival. Central to this process is organization of photosynthetic machinery that captures energy from light. This vital process provides energy to fix carbon into organic matter that is essential for plant growth and development.

Light quality and quantity is sensed by a suite of specialized photoreceptors. An established class of these receptors are the red (R) and far-red (FR) light absorbing phytochromes that have an important role driving the switch from skotomorphogenic to photoautotrophic growth during seedling de-etiolation (reviewed by [1]). The phytochromes exist in two interconvertible forms. When the inactive-Pr form absorbs R light, it converts to the active, Pfr form and relocates from the cytoplasm into the nucleus. Once in the nucleus, phytochromes induce major changes in gene expression that modify the developmental program [2]. Amongst the phytochrome-mediated changes during this critical time is the promotion of the photosynthetic apparatus assembly, and the production of the photosynthetic pigments, chlorophylls and carotenoids [3]–[5] While chlorophyll production is required for photoautotrophic growth, excessive accumulation of the chlorophyll precursor protochlorophyllide can lead to harmful photo-oxidative damage [6]–[8]. It is therefore critical that this process is stringently regulated. Carotenoids are integral accessory pigments in the light absorbing antenna. In the greening process, carotenoids play an essential photoprotective role minimizing the potentially damaging effects of light on the photosynthetic machinery [9]–[11]. Therefore, to maximize light capture but curtail the potentially damaging effects of light over the emerging photosynthetic machinery, the production of carotenoids and chlorophylls has to take place in a tightly controlled and interdependent manner. This might be achieved in part by the circadian gating of isoprenoid pathway genes, that produce both carotenoids and chlorophylls, to ensure coordinated pathway gene expression [12]. However, we do not currently understand how this regulation is influenced by either the regular daily or unexpected variations in external light and temperature.

Earlier studies have shown that bHLH-Phytochrome Interacting Factors (PIFs) not only prevent the over-accumulation of protochlorophyllide in etiolated seedlings [8] but have a broader role in the seedling as repressors of photomorphogenic development [1], [13]. In accordance, a PIF quadruple mutant (*pif1-1;pif3-3;pif4-2;pif5-3*) (*pifQ*) exhibits a constitutive photomorphogenic phenotype in darkness and a global gene expression pattern that resembles R light-grown seedlings [1], [14]–[16]. Concurring with these observations, we previously showed that PIFs repress carotenoid accumulation by down-regulating the expression of the *Arabidopsis thaliana* gene encoding *PHYTOENE SYNTHASE* (*PSY*), the main rate-determining enzyme in the carotenoid pathway [17]. PIF1, a potent *PSY* gene repressor, effects control through direct binding to at least one of the the two G-box (CACGTG) elements found in the *PSY* promoter. Indeed, PIFs have been shown to preferentially target G-box motifs in a range of genes [8], [18]–[21]. Suppression of *PSY* transcription is strongest in etiolated seedlings when PIF are abundant, while exposure to light leads to a depletion in PIF levels and concomitant de-repression in *PSY* expression [17].

PIFs are strongly regulated by light and therefore their activity is conditional on the external light environment. In de-etiolated seedlings growing in light/dark cycles phytochromes control the degradation and re-accumulation of PIF proteins (PIF3, PIF4 and PIF5) generating a daily alternation in the abundance of these transcription factors [22]–[24]. For PIF4 and PIF5, additional temporal control is delivered by the circadian clock that regulates the phase of expression [23], [25]–[27]. As these PIFs are potent regulators of cell expansion in the seedling hypocotyl, this translates to a diurnal rhythm in hypocotyl elongation rate [23], [25]). Recent studies have shown that PIFs also participate in temperature signalling. PIF4, and to a lesser extent PIF5, are required for the promotion of growth in warmer conditions [28], [29]. Elevated temperatures promote the accumulation of phosphorylated PIF4 and enhance PIF4 binding at target promoters [30]–[32]. Indeed, PIF4 occupation at the *FLOWERING TIME* (*FT*) promoter increases with the eviction of H2A.Z nucleosomes by temperature [32].

For a broad range of responses PIFs act antagonistically with the bZIP transcription factor, HY5 [1], [33], [34]. HY5 drives photomorphogenic development by activating genes that promote photosynthetic machinery assembly, photopigment production, chloroplast development, and seedling cotyledon expansion [21], [35], while PIF suppression of these responses is required to maintain skotomorphogenesis [1], [33]. HY5 has been shown to regulate gene expression directly through interaction with “ACE” motifs (ACGT containing elements) that include the Z-box (ATACGTGT), C-boxes (GTCANN), G-boxes, and hybrid C/G (G) and C/A boxes [35]–[38]. Like PIFs, HY5 protein levels are light regulated – but in contrast to PIFs that are light labile, HY5 protein is stabilized by light [1], [39], [40]. Multiple photoreceptors participate in the accumulation of HY5 protein in the light, in part by reducing the nuclear levels of CONSTITUTIVE PHOTOMORPHOGENIC 1 (COP1), an E3 ubiquitin ligase that targets HY5 for proteasome mediated degradation in the dark [39]–[41]. The differential regulation of HY5 and PIF proteins by light means that they exhibit opposing diurnal expression patterns [23], [24], [35], [42].

Whereas PIF4 mediates warm temperature signalling, HY5 has been shown to have a role in cold temperature responses [43]. Here HY5 has been implicated in cold acclimation where it promotes anthocyanin accumulation, ROS production, and regulates a large swathe (∼10%) of cold inducible genes [43]–[45]. HY5 abundance is regulated by temperature. A shift in temperature from 20°C to 4°C elevates HY5 transcript levels and stabilises the HY5 protein through nuclear depletion of COP1 [43].

This study examines the impact of light and temperature on the control of photopigment production by HY5 and PIFs. ChIP, EMSA and transcript analysis established that HY5 and PIF1/PIF4 impart antagonistic regulation to common gene targets through direct binding to the same G-box *cis* element. This dynamic activation-suppression transcriptional module does not act alone, but with the circadian clock to modify the level of rhythmic gene expression. However, abrupt changes in either light or temperature adjusts the equilibrium of HY5 and PIF bound to target promoters altering the transcriptional response. In this way the expression of photopigment biosynthesis genes can be revised by external signals.

## Results

### HY5 is required for light induction of *PSY* expression, and photopigment synthesis

**Figure 1:**
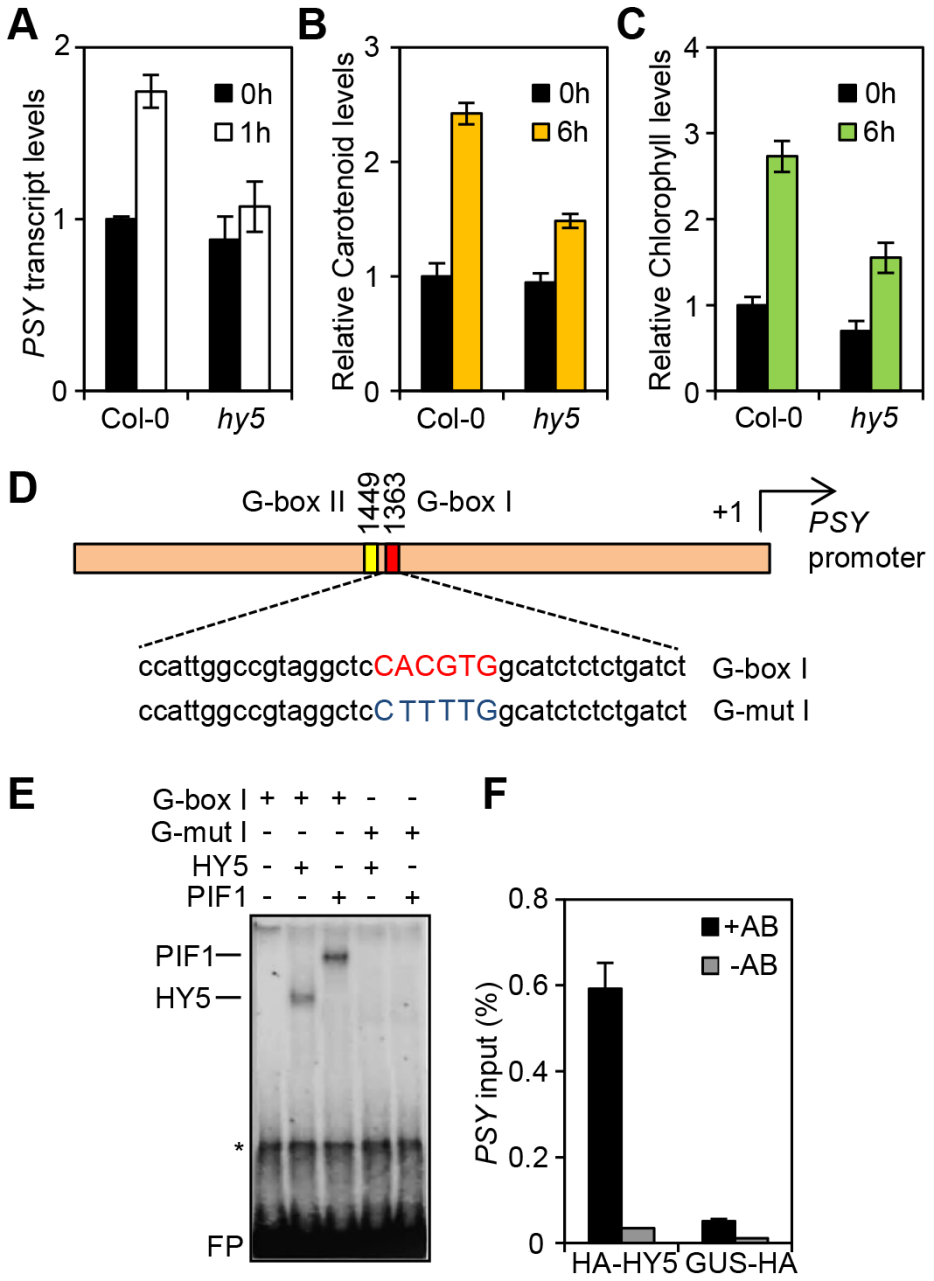
HY5 is a positive regulator of carotenoid and chlorophyll biosynthesis and controls *PSY* gene expression by promoter binding. (**A**) *PSY* expression in 3-day old Col-0 and *hy5-215* seedlings grown in the dark (0 h, black columns) or after 1 h Red light illumination (1 h, white columns). Expression was measured in biological triplicates by qPCR. *PSY* expression levels were normalized against *APT1* levels and expressed relative to Col-0 dark (0 h). Error bars represent± Standard Error (SE). (**B–C**) Carotenoid and chlorophyll content in 3-day old Col-0 and *hy5-215* seedlings. Photopigment content was measured in the Dark (0 h) or after 6 h R light illumination (40 µmol m^−2^ s^−1^). Error bars represent ±SE of biological triplicate sets. Levels (µg/g fresh weight) are expressed relative to Col-0 Dark sample. (**D**) Schematic representation of the *PSY* promoter with the location of the two G-box motifs. Under the diagram, the sequence of the G-box I probe and the mutant G-box I probe (G-mutI) labelled with ^32^P for Electrophoretic Mobility Shift Assays (EMSA) is indicated. (**E**) HY5 and PIF1 bind *in-vitro* to the G-box I in the *PSY* promoter in EMSA assays. For the assay, radiolabelled probes were incubated with TnT produced PIF1 and HY5 proteins. G-box I or G-mut I probes were used as indicated. Asterisk (*) corresponds to a TnT non-specific band and FP stands for free probe. (**F**) HY5 binds *in-vivo* in Chromatin immunoprecipitation (ChIP) assays to the G-box region in the *PSY* promoter. ChIP assays were carried out using a 35S:: HA-HY5 line with antibodies against the HA-tag. A 35S::GUS-HA line was used as a control (+HA antibody samples). A second set of samples were processed without antibody and used as negative controls (-HA antibody). Immunoprecipitated DNA was amplified by qPCR using primers against the G-box region in the *PSY* promoter (see Table S1). For the assay, plants were grown for 1 week at 22°C in white light (80 µmol m^−2^ s^−1^) and then transferred to 1 week growth under Red light (40 µmol m^−2^ s^−1^). Error bars indicate ± SE of biological triplicates.

Our earlier work showed that PIFs restrict the accumulation of carotenoids during de-etiolation, in part by negatively regulating *PSY*, a rate limiting step in the carotenoid biosynthetic pathway [17]. We demonstrated that sequential removal of PIFs leads to incremental rises in *PSY* mRNA levels, illustrating that PIFs act redundantly to suppress *PSY* expression. This data also suggested the existence of a *PSY* activator that becomes more effective with PIF depletion. As HY5 is known to act in opposition to PIFs for a number of photomorphogenic responses [7], [14], [37], [46], we tested whether HY5 fulfilled this role by quantifying *PSY* gene expression in the *hy5* mutant (*hy5-215*) during red light-triggered de-etiolation (Figure 1A). Our data confirmed that *hy5* perturbs *PSY* accumulation following exposure to light. Likewise, red light induction of carotenoid and chlorophyll levels is severely attenuated in the *hy5* mutant (Figure 1B–C). This response contrasts with that in *pif1* and *pifQ* mutants, where *PSY* mRNA and photopigment levels are significantly elevated in dark and red light illuminated seedlings [17]. Because mutants defective in the HY5 homologue HYH did not exhibit photopigment accumulation defects under our conditions, we therefore focussed our analysis on HY5 (Figure S1). Our data illustrate that HY5 and PIFs have opposing roles in the regulation of *PSY* expression and carotenoid and chlorophyll biosynthesis during seedling de-etiolation.

### HY5 binds to the *PSY* promoter through a G-box element

Since HY5 and PIFs confer antagonistic regulation to *PSY*, we wanted to establish whether this was mediated at the *PSY* promoter. Previous chromatin immunoprecipitation (ChIP)-chip analysis indicated that HY5 binds to promoter regions that carry different types of the generic ACE motif (CACGT) including CA hybrid (GACGTA), CG hybrid (GACGTG), Z-boxes (ATACTGTGT), as well as G-boxes (CACGTG), the main recognition motif for PIFs [17], [21], [35], [37]. The *PSY* promoter contains two G-boxes (I and II), separated by 80 base pairs, but not CG, CA or Z type motifs (Figure S2 and Figure 1D). We therefore reasoned that the opposing actions of HY5 and PIF1 on *PSY* expression could be mediated through the same regulatory G-box element. This we tested using an *in-vitro* electrophoretic mobility shift assay (EMSA). Previously we showed that PIF1 did not bind *in-vitro* to G-box II [17], therefore our analysis is focussed exclusively on G-box I. Consistent with our previous results we detected PIF1 binding to a template encompassing the *PSY* G-box I (Figure 1D, E) [17]. In the same assay, we were able to demonstrate that like PIF1, HY5 bound to a labelled probe containing this G-box, but not a mutated version (G-mut I) where the G-box had been disrupted (Figure 1E).

To establish if we could verify our findings *in-vivo* we conducted a ChIP assay using the 35S::HA-HY5 lines used in a previous study that identified genome wide the targets of HY5 regulation [35]. After immunoprecipitation of protein-DNA complexes using the HA antibody, enriched DNA sequences were amplified by qPCR using primers bordering the *PSY* promoter G-box region. As shown in Figure 1F, in contrast to the 35S::GUS-HA and the no-antibody controls, we detected significant enrichment of *PSY* promoter sequences containing G-box I in 35S::HA-HY5 samples. These results together with our *in vitro* analysis suggest that HY5 regulates *PSY* transcription through direct binding to the G-box region of the promoter.

### HY5 enhances carotenoid and chlorophyll pigment synthesis at cooler ambient temperatures

**Figure 2:**
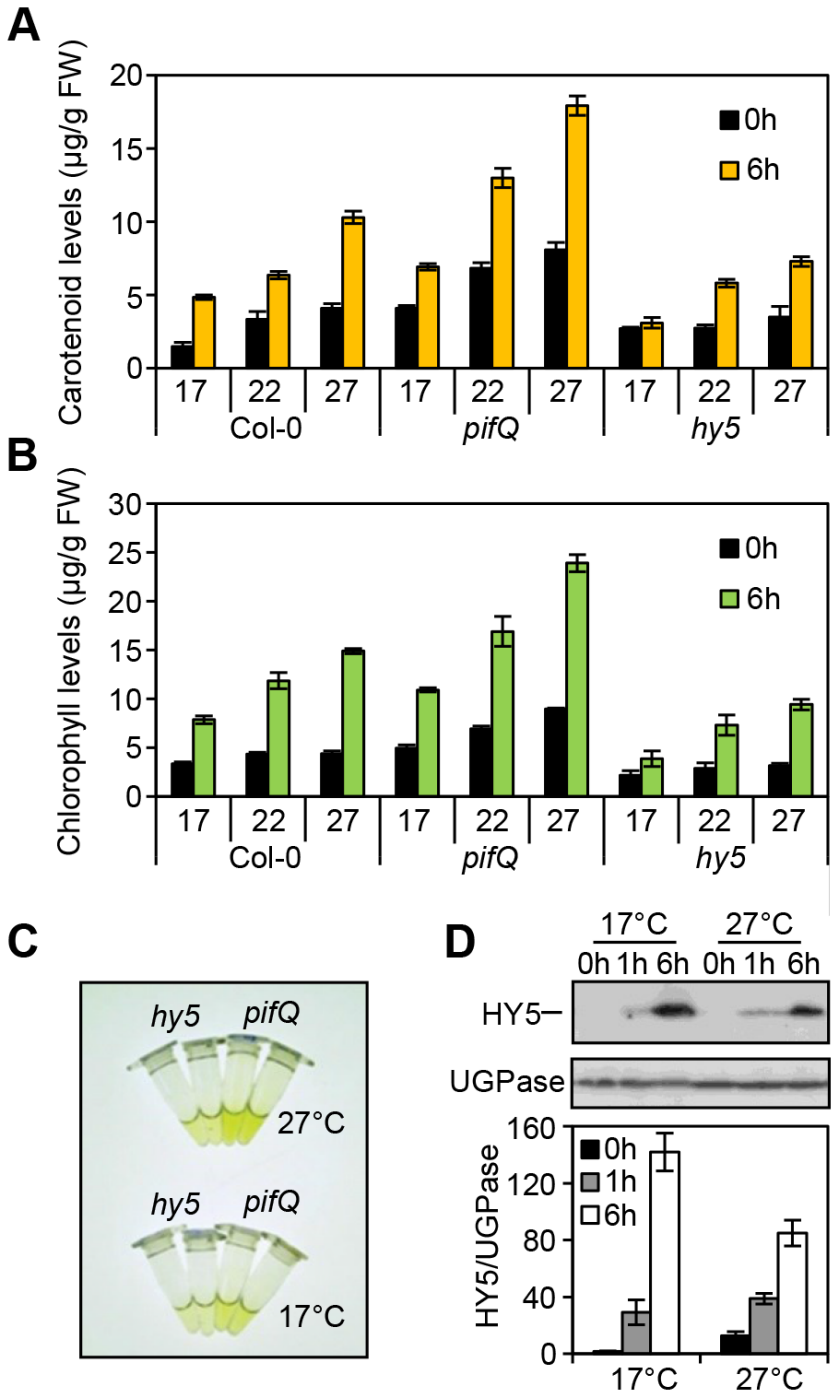
Photosynthetic pigment accumulation is temperature sensitive and dependent on HY5 and the PIFs. **(A–B)** Carotenoid and chlorophyll accumulation in Col-0, *hy5-215* and *pifQ* (*pif1-1 pif3-3 pif4-2 pif5-3*) 3 day-old seedlings grown at different temperatures (17, 22 and 27°C) in the dark (black columns) or after 6 h Red light illumination (40 µmol m^−2^ s^−1^) (yellow or green columns). For measurements seedlings were kept for two days at 22°C and 1 day at the indicated temperature in darkness. On day 3 they were subjected to red light treatment (for 6 h). The control set was kept in darkness (0 h time point). Graphs represent the results for biological triplicates sets. Error bars indicate ± SE. **(C)** *hy5-215* and *pifQ* accumulate different levels of pigments. Illustrative picture of the chlorophyll accumulation response in *hy5-215* compared to the *pifQ* at 17°C and 27°C. Plant material and chlorophyll extraction was conducted as indicated in (A–B). **(D)** HY5 protein accumulates to moderately higher levels at 17°C than at 27°C in response to light. Immunoblot of HY5 protein in 35S::HA-HY5 seedlings grown in darkness for 5 days at 17°C and 27°C before illuminating with Red light (40 µmol m^−2^ s^−1^) for 1 h and 6 h. Quantification of protein levels was conducted relative to the UGPase signal. Error bars represent ± SE of three biological repeats.

As PIFs (e.g. PIF4 and PIF5) and HY5 have been implicated in temperature signalling we wanted to assess whether this action extended to carotenoid and chlorophyll regulation [28]–[31], [43]–[45], [47]–[51]. We found that in both etiolated and red light exposed wild type seedlings carotenoid and chlorophyll levels rose incrementally with temperature (17°C, 22°C and 27°C) (Figure 2A–C). Compared to the wild type, the *pifQ* mutant had constitutively elevated carotenoid and chlorophyll levels under all conditions, but still remained responsive to temperature (Figure 2A, B). This suggests that while specific PIFs (e.g. PIF4) may operate at warmer temperatures, the collective action of PIFs maintain control of carotenoid and chlorophyll levels over a temperature range [28], [30]–[32], [47], [49] In contrast, *hy5* suppressed red light-induction of carotenoid and chlorophyll levels (Figure 2A–C). While the effects of *hy5* were apparent across temperatures, its impact was most marked in the cooler 17°C conditions.

We next measured HY5 protein levels in etiolated and de-etiolating seedlings at 17°C and at 27°C. Concurring with published work, our data show that HY5 transcript and protein levels increase steadily following exposure to 1 h then 6 h red light (Figure 2D, Figure S3) [39]. In the dark, HY5 transcript and protein levels were low and not particularly affected by temperature in our study range. However, we detected higher levels of HY5 transcripts and protein at 17°C when compared to 27°C, after exposure to -red light (Figure 2D, Figure S3). Thus, HY5 - abundance correlates well with both the light and the temperature requirement for HY5 control of photopigment levels (Figure 2A–C).

### HY5 and PIFs regulate a subset of photosynthetic pigment genes through a common mechanism

To establish whether G-box element convergence represented a generic mechanism through which HY5 and PIF operate, we tested the impact of *pifQ* and *hy5* mutations on the expression of other genes central to carotenoid or chlorophyll biosynthesis and function with G-box elements in their promoters. These genes were chosen from genome wide transcriptional analyses specific to PIFs or HY5 [14], [21], [35], [44]. To rule out binding to other promoter elements, the selected genes did not possess any of the alternative high affinity binding sites for HY5: C, Z or C/A, G/A or G/A boxes (Figure S1). For carotenoid biosynthesis, in addition to *PSY*, we selected *VIOLAXANTIN DE-EPOXIDASE* (*VDE*) that is involved in the conversion of violaxanthin to zeaxanthin for optimal photoprotection in the xanthophyll cycle [52]. Chlorophyll biosynthesis genes are represented by *PROTOCHLOROPHYLLIDE OXIDOREDUCTASE C* (*PORC*), that phototransforms endogenous protochlorophyllide to chlorophyllide, *GENOMES UNCOUPLED 5* (*GUN5*) that encodes the ChlH subunit of Mg-chelatase, a key enzyme in the chlorophyll branch of the tetrapyrrole pathway [53], [54], and the Photosystem I *LIGHT-HARVESTING COMPLEX 4 (LHCA4)* [55], [56].

**Figure 3:**
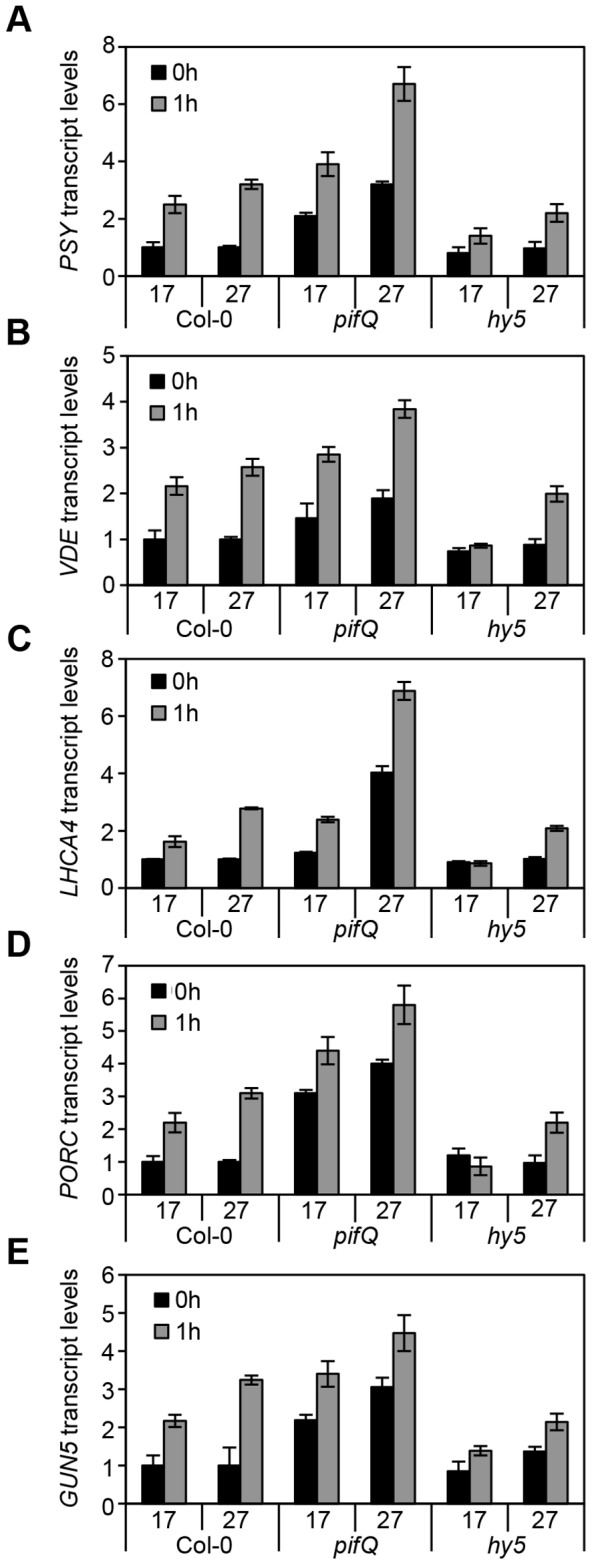
Expression levels of genes related to photosynthetic pigment accumulation at 17°C and 27°C in Col-0, *pifQ* (*pif1-1 pif3-3 pif4-2 pif5-3*) and *hy5-215*. (**A–E**) Gene expression measured by qPCR for *PSY, VDE, LHCA4, PORC* and *GUN5* in 3-day old etiolated seedlings. Samples were grown in the dark for 2 days at 22°C and then moved to 1 day at the indicated temperature, before illumination for 1 h with R light (1 h, grey columns) or maintained in darkness (0 h, black columns). Expression is represented relative to Actin7. Measurements were taken for biological triplicates. Error bars represent ± SE.

**Figure 4:**
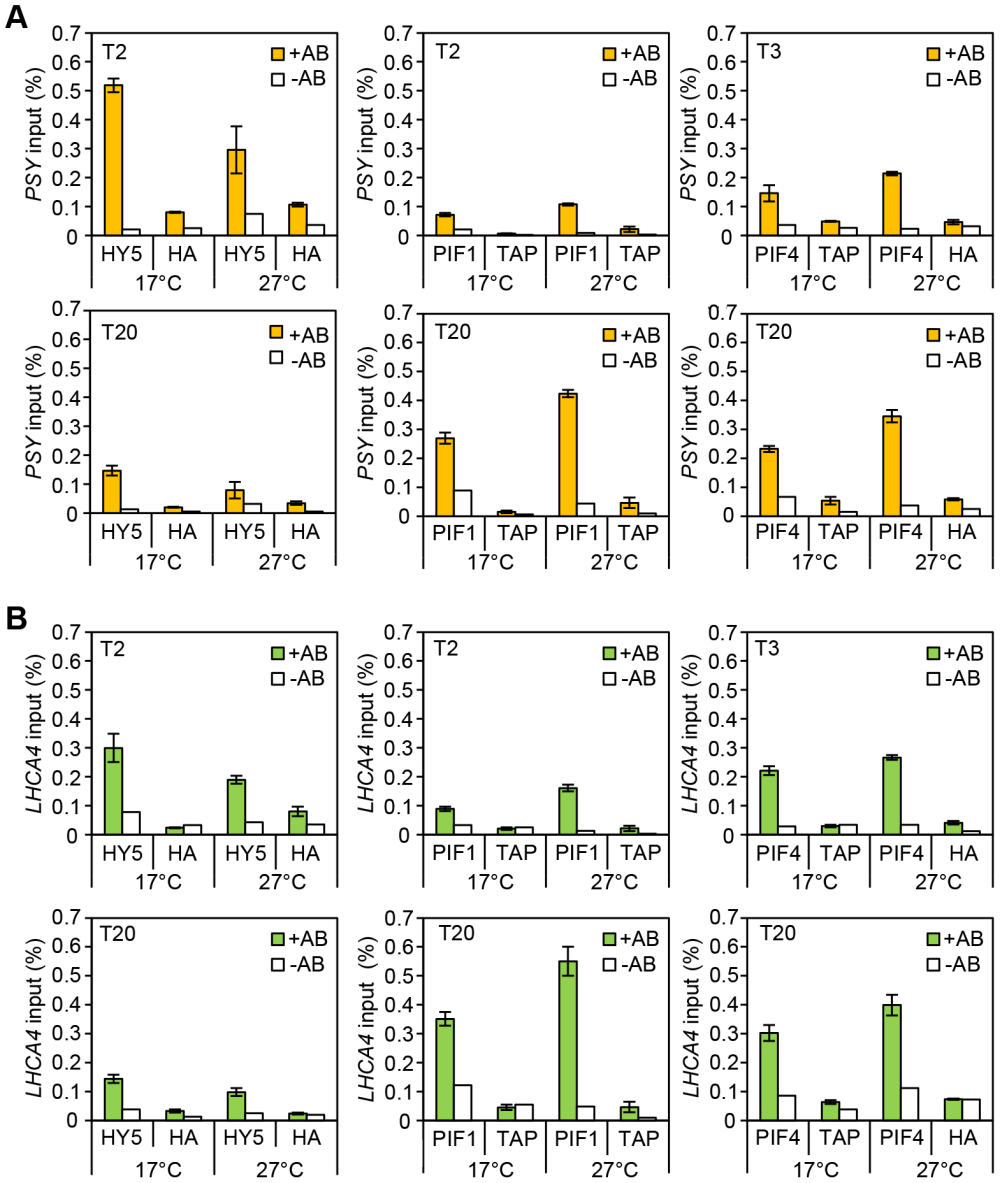
Chromatin immunoprecitation assays for 35S::HA-HY5, 35S::TAP-PIF1 and 35S::PIF4-HA grown in red-diurnals at 17°C and 27°C. Two week old seedlings grown for one week in white diurnal cycles (12 h light/12 h dark, 80 µmol m^−2^ s^−1^) at 22°C, followed by one week under Red diurnal cycles (12 h light/12 h dark, 40 µmol m^−2^ s^−1^) were used for the experiment. On the last day, samples were taken 2 or 3 h after the lights came on (T2/T3) and 8 h after the lights were off (T20). ChIP was carried out using antibodies against the tag (anti-HA or anti-MYC). 35S::GUS-HA (labelled HA) or a 35S::GFP-TAP (TAP) lines were used as controls. A non-antibody control sample was processed in parallel in each case (white bars). Immunoprecipitated DNA was analysed by qPCR using specific primers covering the G-box containing region in the promoters for the indicated genes (see Table S1 for primer information). The assay was carried out in triplicates. Error Bars represent ±SE. (**A**) ChIP results for *PSY*. (**B**) ChIP results for *LHCA4*.

The transcript levels of these genes tightly correlated with the carotenoid and chlorophyll accumulation data (compare Figure 3 with Figure 2A, B). Consistently, mRNA levels were elevated in dark or light-grown *pifQ* at 17°C and 27°C, while *hy5* led to reduced red light induction of each target gene, particularly at 17°C. To establish whether HY5 and PIFs regulate this suite of genes through common G-box elements we conducted ChIP analysis with lines expressing 35S::HA-HY5, 35S::PIF1-TAP and 35S::PIF4-TAP. In these experiments seedlings were grown in more natural 12L:12D cycles sampling at 2 or 3 h post dawn (T2, T3) or 8 h post dusk (T20) (Figures 4 and Figure S2). Levels of binding to the *VDE* promoter, were very low which prevented detailed analysis (data not shown). However, we detected enrichment of HY5, PIF1 and PIF4 relative to controls, at G-box containing regions of *PSY*, *LHCA4, PORC* and *GUN5* promoters. G-box *cis* element convergence therefore appears to represent a common mechanism through which HY5 and PIFs regulate some carotenoid and chlorophyll biosynthetic genes (Figure 4).

### PIF1 action is temperature regulated

Consistent with the more prominent role for HY5 at cooler temperatures (Figure 2) we detected an enrichment of HY5 at each promoter at 17°C compared to 27°C (Figures 4, and Figure S4). This may reflect a change in HY5 protein abundance that is detectable after exposure to 6 h of red light (Figure 2D). Previously we showed that heat leads to an accumulation of phosphorylated PIF4. This process appears to be extremely temperature sensitive as stepped increases in temperature lead to incremental rises of modified forms of PIF4 (Figure S5 A). We also observed increased binding of PIF4 to *PSY, LHCA4* and *GUN5* at 27°C compared to 17°C, but this was relatively modest [30] (Figures 4 and Figure S4). Interestingly, we detect slightly elevated protein levels and enhanced PIF1 promoter binding at 27°C suggesting that PIF1 is also temperature-regulated (Figures 4, Figure S4 B and Figure S5). In support of this notion our genetic data illustrates that like *pif4pif5*, *pif1* has a greater impact on carotenoid and chlorophyll accumulation at 27°C compared to 17°C (Figure S6). Our results show the relative binding, particularly of HY5 and PIF1, at the G-box motifs in the promoters of several photopigment genes varies according to the ambient temperature regime.

### HY5 and PIFs exhibit opposite diurnal shifts in promoter binding

Analysing binding during the daytime compared to the night allowed us to establish whether the documented diurnal changes in HY5, PIF4 and PIF1 abundance [16], [22]–[24], [35], [42] led to corresponding changes in binding. The levels and the proportion of HY5 bound to *PSY, LHCA4, PORC* and *GUN5* promoters was greater at T2 than T20 (Figures 4, Figure S4 and Figure S5 C). PIF1 had the converse response with less bound at T2 (when PIF1 is less abundant) than T20 (Figures 4, Figure S4 and Figure S5 B). This trend was also evident, but less marked, for PIF4. This may reflect less dramatic diurnal changes in 35S::PIF4, the line used in this study, compared to 35S::HY5 and 35S::PIF1 (Figures 4, Figure S5 D and Figure S4). We then conducted EMSA to test whether a change in protein abundance is sufficient to drive a switch in binding to the G-box promoter fragment. In this assay the “primary” protein (either HY5 or PIF1) was incubated with a fixed amount of the G-box I probe and then the “challenge” protein was added at increasing concentrations (Figure S7). We could not detect a switch in binding when an equimolar amount of either “challenge” protein was added. However, binding was detected with increased concentrations of “challenge” protein, with a correlative decrease in “primary” protein binding. This indicates that when provided in excess HY5 or PIF1 can indeed prevent the other protein from binding.

### HY5 and PIFs regulate photopigment biosynthetic genes in cooperation with the circadian clock

**Figure 5:**
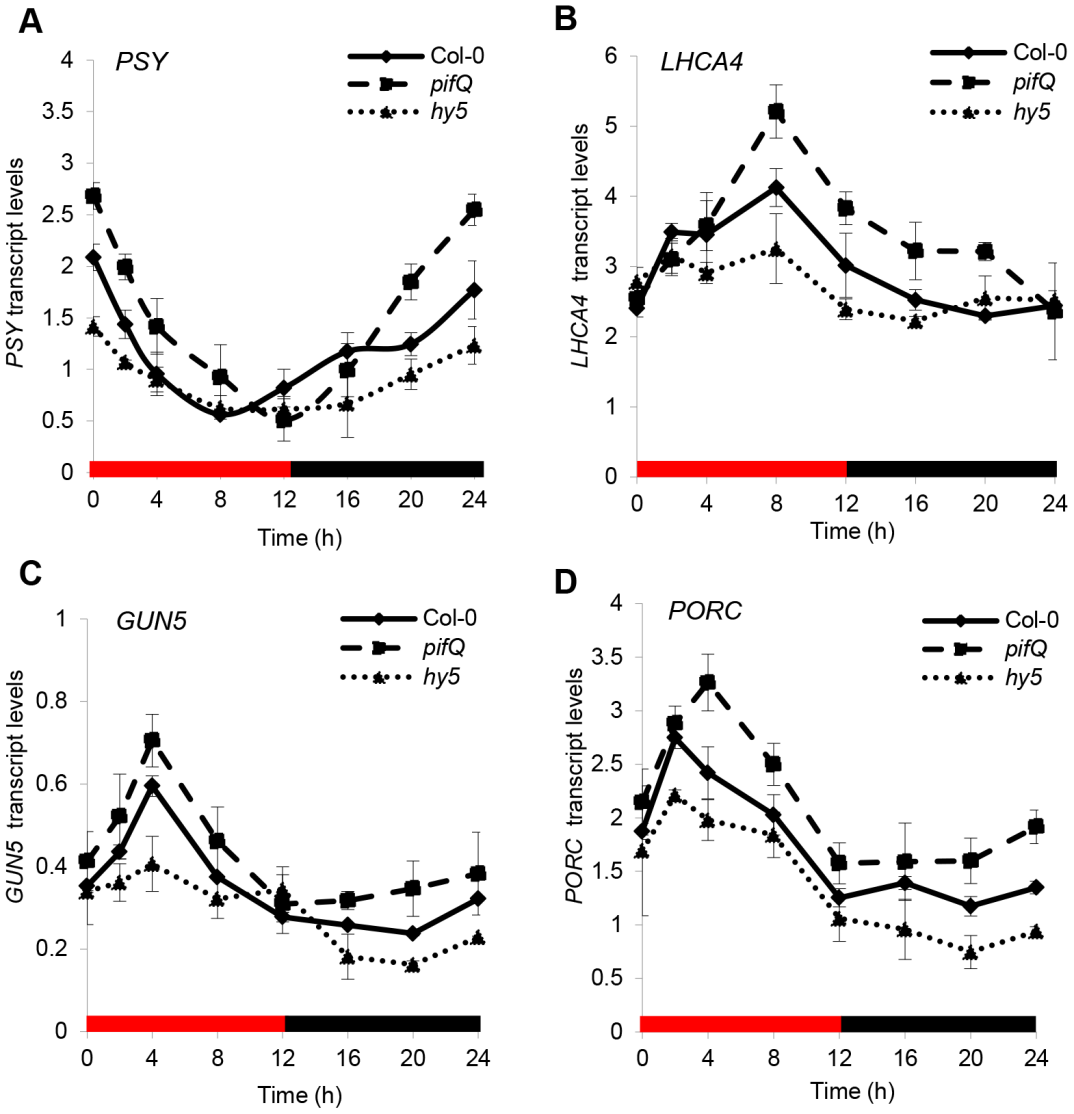
Red diurnal expression patterns of photopigment genes targets of PIFs and HY5. **(A–D)** Expression by qPCR of *PSY* **(A)**, *LHCA4* **(B)**, *GUN5* **(C)** and *PORC* **(D)** in Col-0, *pifQ* (*pif1-1 pif3-3 pif4-2 pif5-3*) and *hy5-215* backgrounds. Plants were grown for one week under white diurnals (12 h light/12 h dark, 100 µmol m^−2^ s^−1^) at 22°C and then were transferred for 7 days to Red diurnal cycles at 17°C before sample collection at the indicated time points. Gene expression was analysed by qPCR. Black bars represent the dark period and red ones the illuminated times. Error bars represent ± SE of biological triplicates.

Through a red/dark cycle at 17°C, wild type expression profiles of *PSY*, *LHCA4, GUN5* and *PORC* are rhythmic with differing phases of expression, suggesting underlying regulation by the circadian oscillator (Figure 5). The *hy5* and *pifQ* mRNA profiles appear to approach or on occasion converge with the wild type at distinct phases in the cycle, suggesting that HY5 and PIF control of this gene set is gated by the clock (Figure 5). The contrasting day/night shifts in HY5 *vs* PIF1 binding to target promoters (Figure 4, Figure S4) inferred that the *hy5* mutation should be more effective during the daytime and *pifQ* more potent at night. Unexpectedly, we found that this is not the case, as both *hy5* and *pifQ* cause a shift in transcript levels during the day and the night (Figure 5). This indicates that during a diurnal cycle there is not a simple relationship between our recorded changes in promoter binding and the transcriptional response. It also indicates that HY5 and PIFs operate through the light/dark cycle to regulate this gene set. Interestingly, *HY5* transcript levels are elevated in the *pifQ* mutant, while *PIF4* and *PIF5* (but not *PIF1* or *PIF3*) mRNA levels are raised in *hy5* at T2 and T20 (Figure S8). Such cross-regulation is predicted to increase the amplitude of expression in *pifQ* and conversely, reduce the amplitude in *hy5* (Figure 5). In support of this notion, at least for *PSY*, *LHCA4* and *GUN5* the data show amplitude differences in *pifQ* compared to *hy5* (Figure 5).

The data presented suggest that HY5 and PIFs regulate gene expression in cooperation with the clock. This is further supported by the observation that the cross-regulated genes, *HY5, PIF4* and *PIF5* exhibit strong diurnal regulation, whereas *PIF1* and *PIF3* are not rhythmic and are not subject to cross-control (Figure S8 B) [22], [24]. Collectively, our analysis shows that HY5 and PIFs do not exhibit strong diurnal shifts in activity, but operate with the circadian clock to moderate rhythmic gene expression through the light/dark cycle.

### Abrupt changes in HY5 and PIF protein abundance control binding to target gene promoters

**Figure 6:**
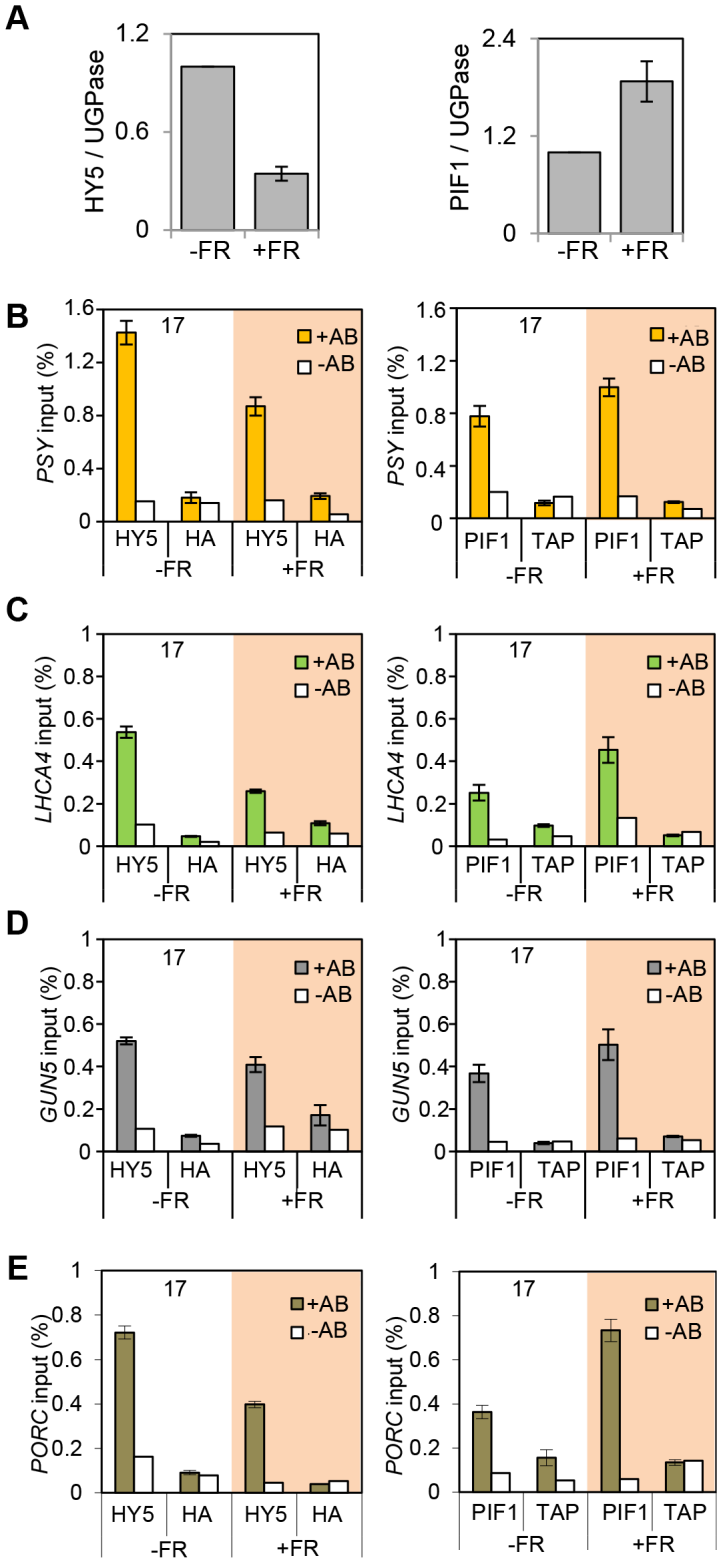
End of Day FR (EOD-FR) effect over 35S::HA-HY5 and 35S::TAP-PIF1 binding to G-box regions in the promoters of genes related to carotenoids and chlorophyll accumulation at 17°C. (**A**) Protein content quantification relative to the signal of UGPase for 35S::HA-HY5, 35S::PIF1-TAP at time 15 h (T15), in samples treated with (+FR) or without (-FR) an EOD saturating FR light pulse (3000 µmol). Triplicate Immunoblots were carried out using 2 week old seedlings. Seedlings were grown for one week in 12 h light/12 h dark white diurnal cycles at 22°C and then transferred to 17°C, 12 h light/12 h dark Red diurnal cycles. Protein was extracted at T15 and quantified against UGPase signal. (**B–D**) Chromatin Immunoprecipitation assay for *PSY* (**B**), *LHCA4* (**C**), *GUN5* (**D**) and *PORC* (**E**) G-box regions in 35S::HA-HY5 and 35S::PIF1-TAP plants treated with (+) or without(−) an EOD FR pulse at T15. +AB indicates samples treated with antibody, -AB stands for no antibody controls. Plants were grown as indicated in (**A**) and in material and methods. The HA- and MYC- controls and ChIP procedure were described in Figure 4. Error bars represent ± SE of biological triplicates.

**Figure 7:**
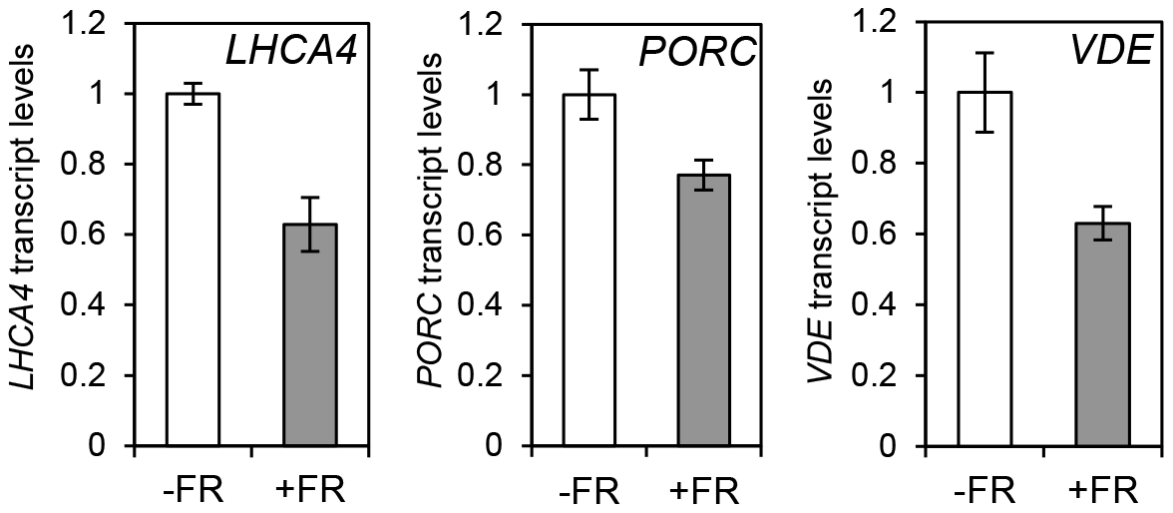
Photopigment gene expression response to an End of Day (EOD)-Far Red (FR) light treatment in Col-0. Expression levels for *LHCA4*, *PORC* and *VDE* in Col-0 plants treated without (−FR) or with (+FR) a saturating FR pulse (3000 µmol) at the end of the day (EOD) and collected at T15. Samples were grown as in Figure 6, and used for gene expression measurements by qPCR. Levels are expressed relative to Col-0 (-FR) T15 sample and normalized against *ACT7* expression. Error bars represent ± SE of biological triplicates.

The observation that the relative action of HY5 and PIF did not change during a diurnal cycle appeared to be at odds with our finding that light or temperature stabilisation of HY5 led to concomitant rises in binding activity and gene regulation (Figures 1, 3, 4 and Figure S4). This suggested that a sudden or sizeable increase in HY5 levels relative to PIFs may activate gene expression. To test this hypothesis we next manipulated HY5 and PIF1 levels *in vivo* to establish whether we observed corresponding changes in promoter binding and gene regulation. In these experiments plants were grown in 17°C diurnal cycles and protein levels were measured 3 h into the darkness in controls or following an end-of-day FR (EOD-FR) pulse which deactivates phytochrome [57]. This treatment led to a concurrent rise in PIF1 protein levels and fall in HY5 protein levels (Figure 6 A). In response to EOD-FR we observed correlative alterations in HY5 and PIF1 binding to the *PSY, LHCA4*, *GUN5* and *PORC* promoters (Figure 6 B–E). We then tested whether EOD-FR led to predictable changes in gene regulation. *PSY* and *GUN5* were unresponsive to the EOD-FR treatment (Figure S9). This result was unsurprising for *PSY*, as the EOD-FR timing coincided with the phase in which PIFs and HY5 are not fully engaged in transcriptional regulation. Likewise, the contribution of PIFs and HY5 to *GUN5* transcriptional regulation was relatively low when compared to the other genes (Figure 5). Nonetheless, for *LHCA4, PORC* and *VDE*, the EOD-FR treatment elicited a decrease in transcript levels (Figure 7), a response that is consistent with the observed changes in PIF *vs* HY5 protein abundance and promoter binding.

### HY5 regulates photosynthetic capacity at cooler temperatures

**Figure 8:**
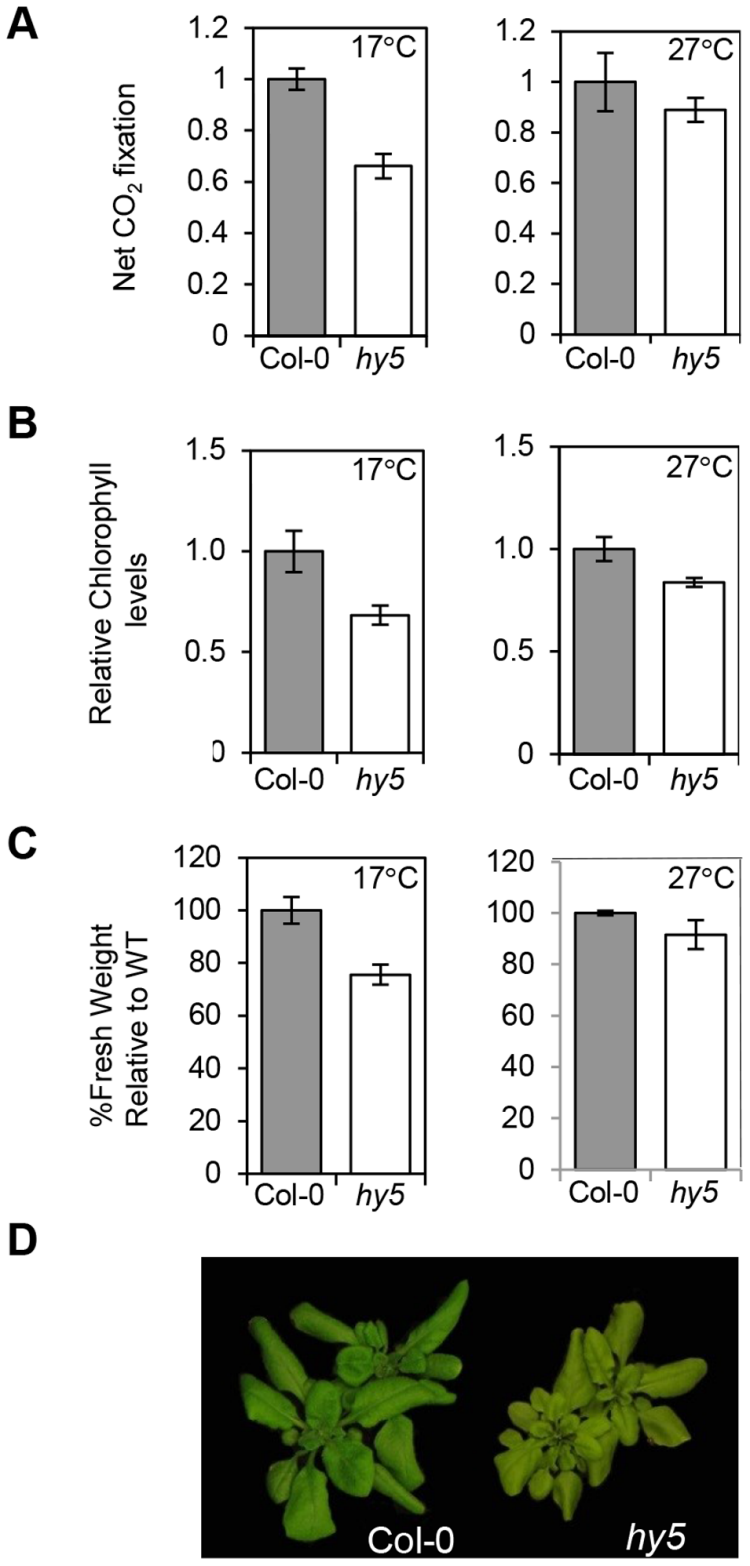
HY5 modulates photosynthetic acclimation efficiency at low temperatures. **(A)** CO_2_ net flux measurements for Col-0 and *hy5-215* acclimated to 17°C or 27°C. Plants were grown for 3 weeks under 12 h light/12 h dark white light cycles at 22°C and transferred for 7 days to same photoperiod cycles at 17°C or 27°C. CO_2_ assimilation was measured in parts per million (ppm). Flux was calculated per unit area (m^2^) and results expressed relative to Col-0. Measurements were conducted in duplicates for two independent sets of 48 plants. Error bars represent ± SE. **(B)** Chlorophyll content for plants used in the CO_2_ assimilation experiment. Chlorophyll was extracted for 12 randomly selected plants. Plants were grown as described in (A). Values were calculated in µg/g fresh weight and expressed relative to Col-0. Error bars represent ± SE. **(C)** Fresh weight difference for Col-0 and *hy5-215* 17°C and 27°C acclimated plants. Weight was measured in grams and result is expressed as a percentage of Col-0 (assigned value of 100%). Bars represent SE of two sets of 48 plants. **(D)** Pictures of representative Col-0 and *hy5-215* plants acclimated to 17°C and used in the experiments described (A–C).

Our data illustrate that HY5 acts antagonistically with PIFs to control photosynthetic pigment genes through a common *cis* element, particularly at cooler temperatures. As this work was conducted in seedlings we wanted to establish the significance of this regulation in older plants. We tested this by measuring photosynthetic capacity in WT and *hy5* adult plants (grown in 12L:12D) shifted to either 17°C or 27°C from 22°C. The *hy5* mutant had lower chlorophyll levels and CO_2_ net flux m^−2^ s^−1^ compared to WT, and this difference is more marked at 17°C vs 27°C (Figure 8 A–B). Our previous studies of *pifQ* demonstrated that PIFs also influence the accumulation of carotenoids and chlorophylls under photoperiodic conditions [17]. It therefore appears that the HY5 and PIF driven transcriptional switch is important for controlling the production of photosynthetic pigments beyond the seedling stage. The temperature dependence of the *hy5* mutation on chlorophyll levels and CO_2_ uptake was evident seven days following the transfer from 22°C to either 17°C or 27°C. This indicates the process is dynamic and that HY5 is required to actively maintain chlorophyll content in response to changing external light and temperature signals. The impact of *hy5* on carbon assimilation may have consequences for growth as fresh weight is markedly reduced in *hy5* mutants at 17°C but not at 27°C (Figures 8 C and D).

## Discussion

### HY5 and PIFs co-regulate photosynthetic genes at common G-box motifs

Despite the knowledge that light and temperature are among the most relevant environmental signals modulating photosynthetic pigment production, there is no integrative view on how these adjustments are achieved. We have shown that by responding to external light and temperature signals, HY5 opposes PIF action to deliver environmental control of common target genes involved in photosynthesis and photoprotection. This dual control system appears to operate in conjunction with the circadian oscillator to adjust levels of rhythmic photosynthetic gene expression.

Our previous work, and that of others, identified the PIFs as negative regulators of chlorophyll and carotenoid accumulation [8], [17], [51], [58]. However, optimization of these essential responses would require an additional regulator that acts antagonistically to PIFs. Here we have shown that HY5 fulfils this role as a positive regulator of photosynthetic pigment synthesis (Figure 1). Our earlier research demonstrated that PIFs control the expression of *PSY*, a rate limiting step in carotenogenesis. This is achieved by directly binding to G-box motifs in the *PSY* promoter [17]. Previous genome wide gene expression and ChIP-CHIP analyses highlighted an overlap in PIFs and HY5 gene targets and identified G-boxes as potential HY5 targets [7], [35], [36], [44], [59]. We have shown *in-vitro* and *in-vivo* that HY5 binds to the same G-box containing region targeted by PIF1 in the *PSY* promoter (Figure 1). Our data indicates that HY5 and PIFs act antagonistically to control *PSY* gene expression via a common *cis* element. This regulation is not confined to *PSY*, as we presented evidence that other central carotenoid and chlorophyll pathway genes are regulated through the same dual input mechanism. HY5 and PIFs do not always act antagonistically. In the case of anthocyanin biosynthesis, PIF3 and HY5 both positively regulate the pathway by activating transcription of the same biosynthetic genes by binding to distinct *cis*-promoter elements (G-box and another ACE motif respectively) [19]. In this instance PIF3 binding is facilitated by the presence of HY5, suggesting cooperativity of action. However, our analysis of photopigment gene regulation, together with a recent study focussing on *REACTIVE OXYGEN SPECIES* genes, indicates that when HY5 and PIF act in opposition, G-box convergence is a common mechanism [7].

### Coaction of HY5 and PIF antagonists fine tune rhythmic photopigment gene expression

Light has opposing effects on HY5 and PIF levels and/or activity [1], [34], [60], [61]. HY5 protein accumulates in the light and is degraded in the dark [39], [41]. In contrast, PIFs accumulate in the dark to promote dark development, and light induces rapid phytochrome dependent phosphorylation, degradation and deactivation [22], [60], [62]–[64]. Accordingly we established that HY5 promotes the expression of *PSY, VDE, PORC, LHCA4* and *GUN5* genes following exposure to light, while PIFs strongly suppress the transcription of these genes in etiolated seedlings (Figure 3).

In diurnal cycles we showed that the levels of HY5, PIF1 and PIF4 bound to common G-box elements correlated with times that these proteins are abundant (Figures 4, Figure S4 and Figure S5). Furthermore, *in vitro* EMSA assays demonstrated that binding of either HY5 or PIF1 to the G-box region of the *PSY* promoter was reduced when the opposing challenge protein was provided in excess (Figure S7). This, analysis illustrated that the change in relative HY5:PIF promoter binding could at least partly be driven by alterations in protein abundance. However, when we analysed the transcript profiles of wild type plants compared to *hy5* and *pifQ* mutants, there was no obvious correlation between the shift in binding dominance and the relative effectiveness of HY5 and PIFs through the light/dark cycle (Figure 5). A potential explanation is that cross-regulation between HY5 and PIFs may dampen the diurnal swings in response. Such a mechanism has been recently reported by Chen and coworkers[7]. This study showed that PIF1 and PIF3 physically interact with HY5 and its homologue HYH. Through this interaction these antagonistic pairs of transcription factors, PIF1/PIF3 and HY5/HYH, moderate each other's control of ROS-responsive genes. It is therefore possible that this cross-control is a more broadly utilised “buffering” mechanism that dampens antagonistic HY5-PIF responses. Alternatively, an independent regulator such as the circadian clock may be modulating the level or activity of HY5 or PIFs at the promoter. Indeed, we showed that HY5/PIF control of *PSY, PORC, LHCA4* and *GUN5* expression is gated by the circadian clock (Figure 5). These findings are consistent with earlier work that shows that the HY5 protein physically interacts with CCA1, which in turn enhances the binding of HY5 to the LHCB1*1 promoter [65]. It is not yet known how PIFs interface with the oscillator. However, an *in vitro* interaction between PIF3 and TOC1/PRR1 has been reported [66], [67], and *GUN5*, a gene identified as a HY5/PIF target in this study, was previously shown to be directly and negatively regulated by TOC1 [68]–[70]. PRR9, PRR7 and PRR5 are also reported to be negative regulators of chlorophyll and carotenoid biosynthesis [71]. Thus, PIFs may regulate specific gene targets in concert with TOC1/PRR1, other PRRs or their regulators. Further analysis will be required to evaluate these and other possible links between HY5, PIFs and the clock.

### Changes in protein abundance alter HY5/PIF promoter binding

The diurnal switch between HY5- and PIF- dominant promoter binding did not lead to correlative diurnal shifts in transcriptional regulation (Figures 4, 5 and Figure S4). However, a sudden change in HY5 or PIF protein abundance *in-vivo* did elicit a matching alteration in gene expression in *LHCA4*, *PORC* and *VDE* (Figure 7). Supplying an EOD-FR pulse, which deactivates phyB, induced a simultaneous fall in HY5 and rise in PIF1 protein abundance, with correlative alterations in promoter binding and transcript regulation (Figures 6 and 7). These results illustrate that abrupt changes in HY5 or PIF levels can lead to corresponding adjustments in signalling. Sizeable adjustments in HY5 and PIF levels and activity are known to occur in etiolated seedlings following exposure to light, and are predicted in plants exposed to abrupt changes in temperature [1], [28], [29], [35], [41], [43], [61], [62], [72], [73].

### Environmental temperature signals are delivered through HY5 and PIF1

Earlier work illustrated that HY5 is important for cold acclimation responses and enhancement of freezing tolerance [43]–[45]. At 4°C HY5 protein levels were shown to stabilise, particularly in the dark [43]. We have shown that HY5 also has a prominent role at the more moderate 17°C, but here its action depends on light. Exposure of etiolated seedlings to light leads to a rapid depletion in PIF levels and increase in HY5 (Figure 2D) [1], [16], [35], [41], [62]. The sudden change from PIF to HY5 dominance induces a switch from transcriptional repression to activation of target photopigment genes (Figure 3). This switch is more robust at 17°C compared to 27°C.

PIF4 is known to activate genes in a temperature dependent manner [30]–[32], [47], [50], [74]. PIF4 levels and its binding to target promoters are boosted by warm temperature [30]–[32]. In line with these studies we observed enhanced PIF4 enrichment at carotenoid and photosynthetic gene promoters under warmer conditions. Interestingly, PIF1 levels and binding to target promoters is also increased at 27°C compared to 17°C (Figures 4 and Figure S4). Our genetic data also show that PIF1 has a greater influence on carotenoid and chlorophyll levels at 27°C in etiolated seedlings (Figure S6). Thus, in the regulation of photosynthetic pigment synthesis, PIF1 action appears to be temperature dependent. The temperature response is particularly evident in etiolated seedlings as PIF protein levels are very high compared to HY5, and therefore PIF signalling predominates. Our data also show that the *pifQ* mutant has perturbed responses at 17°C and 27°C, suggesting that collectively, PIF1, PIF3, PIF4 and PIF5 operate over a broad temperature range (Figures 2 and 3).

### HY5 maintains photosynthetic capacity at lower temperatures through development

We have shown that HY5 is not only important in developing seedling, but as for PIFs, it continues to regulate photopigment levels in adult plants (Figure 8) [17]. Our data demonstrate that HY5 plays a role in maintaining chlorophyll levels and CO_2_ uptake. Interestingly, our analysis indicates that HY5 has a greater impact in plants transferred (from 22°C) to 17°C compared to 27°C (Figure 8 A–C). This illustrates that maintenance of chlorophyll pool is very dynamic and that HY5 is required to augment chlorophyll synthesis and carbon uptake, particularly when temperatures fall. Furthermore at 17°C the fresh weight of *hy5* is markedly reduced - compared to wild type plants at 17°C but mot at 27°C (Figure 8 C–D).HY5 has been shown to modulate several aspects of plant hormones pathways including auxins, giberellins and absicic acid signal transduction. Therefore, it is likely that alterations in hormone signalling will contribute to *hy5* adult phenotype [34] However, the reduction in photosynthetic capacity observed in *hy5* at 17°C (Figure 8) may also compromise growth.

This paper illustrates that signal convergence of antagonistic regulators HY5 and PIFs at a shared *cis* regulatory element provides an effective mechanism to integrate light and temperature signals. A similar type of control has been reported for the endoreduplication E2Fb and E2Fc transcription factors which antagonistically regulate *DEL1* expression through a common *cis*-element [75], [76]. Differential regulation of E2Fb vs E2Fc protein levels by light, determines relative binding capacity and the level of DEL1 activation [77]. It is therefore possible that the single *cis* element activation-inactivation module is a prevalent signalling mechanism through which external signals can change or fine-tune transcriptional responses.

## Materials and Methods

### Plant material and growth conditions

Columbia-0 (Col-0) wild type and mutants were used for experiments. The mutant alleles corresponded to: *hy5-215, hyh* (GK-57200610-N323769), *pif4pif5* (*pif4-2, pif5-2*), *pif1-1*, *pifQ* (*pif1-2, pif3-3, pif4-2, pif5-2*). Over expressing plants included 35S::PIF4-HA, 35S::HA-HY5 and 35S::TAP-PIF1. All have been previously described [14], [35], [58], [78], [79]. Seeds were surface sterilized, sown in GM-agar media and stratified in darkness for 3 days at 4°C before given a 3 h white light pulse to induce germination. For deetiolation experiments (qPCR and photosynthetic pigment measurements), 3 d-old seedlings were used. Seedlings were kept in the dark for 2 days at 22°C and transferred to the indicated temperature for 1d before exposure to red light (40 µmol m^−2^ s^−1^). For Red Diurnal experiments plants were grown for one week under white diurnals (12 h light/12 h dark, 100 µmol m^−2^ s^−1^) at 22°C and then were transferred for 7 days to Red diurnal cycles at 17°C before sample collection. In the case of ChIP experiments, samples were collected at T2 for PIF1 and HY5 and T3 for PIF4. Times were selected based on moments where the proteins have started to recover following phyB degradation (in the case of the PIFs, that starts at T0) and when levels were comparable among all proteins (in the case of HY5) ([80], [81],[22]).

### Carotenoid and chlorophyll measurements

For the experiments, 3 d-old etiolated seedlings were used and treated as indicated above. Total carotenoids and chlorophylls were extracted and quantified spectrophotometrically as described by[17]. Concentration was expressed per sample fresh weight and measured in biological triplicates.

### RNA isolation and transcript levels analysis by qPCR

For deetiolating experiments quantitative qPCR seedlings were prepared and sown as previously described, and grown for 2 days in the dark at 22°C and 1 day at the indicated temperature before red-light illumination (as indicated above). For diurnal expression analyses, 2 week old seedlings were used. Seedlings were grown under white light 12L:12D cycles (80 µmol m^−2^ s^−1^) at 22°C for one week before being transferred to 17°C red light 12∶12 dark/light cycles (40 µmol m^−2^ s^−1^) for one week and harvesting at the indicated time points. Samples were collected in RNAlater (Sigma). RNA was extracted with RNeasy Plant Mini kit (Qiagen). cDNA synthesis was performed using the SuperScript VILO cDNA synthesis kit (Invitrogen). The qPCR was set up with a liquid handling robot (Tecan Freedom EVO) and qPCR performed with a LightCycler 480 (Roche). All samples were processed in biological triplicates.

### Electrophoretic Mobility Shift Assays (EMSA)

EMSA were conducted as described in [18]. In brief HY5 and PIF1 proteins were produced in an *in-vitro* transcription and translation system (TnT) (Promega) and incubated with a *PSY* promoter fragment generated by annealed oligonucleotides containing the G-box motif labelled with ^32^P-dCTP.

### Chromatin Immunoprecipitation assays

ChIP assays were conducted according to [58]except that 2 week old plants were used for the assay. Unless otherwise stated, plants were grown for one week in 12∶12 white light diurnal cycles at 22°C and moved to one week growth in red 12∶12 diurnal cycles at the testing temperature. Samples were harvested at the indicated time points during the diurnal cycle. For the end-of-day FR experiment, a saturating FR pulse (3000 µmol total) was given at the end of the day and samples harvested 3 h after the pulse. A non-FR treated sample was harvested at the same time point. The sequence of the primers used in these experiments to amplify G-box containing promoter regions of individual genes is shown in Table S1.

### Immunoblots

Total proteins were extracted from 100 mg of tissue in a buffer containing 100 mM Tris-HCl pH 8, 50 mM EDTA, 0.25 M NaCl, 0.7% SDS and 1 mM DTT. Samples were heated at 65°C for 10 minutes and then centrifuged at maximum speed to remove debris. Total protein was quantified by Bradford. 30 µg of total protein were loaded. Samples were run in a 10% SDS gel, followed by wet transfer to nitrocellulose. The HA- or MYC- tag were detected by probing with rat-anti HA horseradish peroxidase HRP coupled HA- antibody (3F10 Roche) or an Anti-MYC mouse antibody (mAb 9E10, Calbiochem) at a 1∶5000 dilution followed by HRP-conjugated anti-mouse antibody at 1∶10,000 dilution. Loading was confirmed by reprobing the membranes with an anti-goat UGP-ase antibody (Agrisera) at 1∶1000 dilution followed by a HRP-conjugated sheep anti-goat antibody (Biorad) at a 1∶5000 dilution. Signal was detected with Amersham ECL kit (GE Health care) according to the manufacturer's protocol. Quantification was performed using Image-J software.

### CO_2_ assimilation measurements and chlorophyll measurements

For adult plant analysis seedlings were sown as indicated in plant material and grown for 1 week under white light 12 h L/12 h D regimes at 22°C. Then plants were transferred to soil and kept under the same photoperiod and temperature for 3 weeks. On week 3, plants were transferred to 12 h L/12 h D regimes at 17°C or 27°C for 7 days before measurements were taken. CO_2_ assimilation was measured over a 1 minute period for 48 plants using a EGM-4 machine (PP Systems) for 1 minute. Readings were taken in ppm and plants photographed and weighted to calculate area, fresh weigh and total CO_2_ flux. The same set of plants was used for fresh weight determination and chlorophyll measurements. For chlorophyll extraction 300 mg of fresh tissue from twelve randomly selected plants were used for individual extractions conducted in triplicates. The whole rosette fresh weight was estimated and chlorophyll extracted by the protocol: http://www.nature.com/protocolexchange/protocols/521. Chlorophyll content was measured spectrophotometrically.

## Supporting Information

Figure S1 *hyh* does not affect photosynthetic pigment accumulation during Red controlled deetiolation. **(A–C)** Carotenoid and chlorophyll accumulation in Col-0 and *hyh* 3 day-old seedlings grown at different temperatures (17, 22 and 27°C respectively) in the dark (black columns) or after 6 h Red light illumination (40 µmol m^−2^ s^−1^) (yellow or green columns). For measurements seedlings were kept for two days at 22°C and 1 day at the indicated temperature in darkness. On day 3 they were subjected to red light treatment (for 6 h). The control set was kept in darkness (0 h time point). Graphs represent the results for biological triplicates sets. Error bars indicate ± SE.
(TIF)

Figure S2 Gene sequences and gene promoter regions under analysis. G-box motifs are highlighted in red.
(PDF)

Figure S3 *HY5* transcript levels at 17°C and 27°C. *HY5* expression was measured in Col-0 seedlings grown in darkness for 5 days at 17°C and 27°C before Red light (40 µmol m^−2^ s^1^) illumination for 1 h and 6 h. Quantification of transcript levels was conducted by qPCR and expressed relative to ACT7 levels. Error bars represent ± SE of three biological repeats.
(TIF)

Figure S4 Chromatin immunoprecitation assays for *PORC* **(A)** and *GUN5* **(B)** G-box regions in 35S::HA-HY5, 35S::TAP-PIF1 and 35S::PIF4-HA backgrounds. Plants were grown as indicated in Figure 4 under Red diurnals (12 h dark/12 h light) cycles at 17 and 27°C. Samples were taken at T2/T3 and T20 and processed in the same way as samples from Figure 4. Error bars represent ±SE of biological triplicates.
(TIF)

Figure S5 **(A)** Higher mobility PIF4 forms accumulate as temperature increases. Immunoblot of 35S::PIF4-HA protein extracted from 6 day old seedlings kept in the dark. Seedlings were grown at the indicated temperature. Immunoblots were carried out with an HA antibody. Loading control indicated by UGPase signal. **(B–D)** Quantification of protein abundance for samples grown in the same conditions as the ones used for ChIP at T2 (or T3 for 35S::PIF4-HA) and T20 time points in Figure 4. Protein was quantified by immunoblots for 35S::PIF1-TAP **(B)**, 35S::HA-HY5 **(C)** and 35S::PIF4-HA **(D)** at 17 and 27°C during the morning (at T2/T3, black bars) and the evening (time T20, grey bars). Antibodies against the tag were used for signal detection (anti -Myc or anti-HA) and relative quantification was carried out against UGPase signal. Error bars represent ±SE of biological triplicate samples.
(TIF)

Figure S6 Carotenoid and chlorophyll accumulation for the *pif4pif5 (pif4-2 pif5-3)* double mutant compared to *pif1*(*pif1-1*) and Col-0 at 17 and 27°C. Plants were grown and processed for pigment extraction as indicated in Figure 2. Error bars represent ±SE of biological triplicate sets.
(TIF)

Figure S7 Simultaneous binding of PIF1 and HY5 to the *PSY* promoter in EMSA. The probe preparation and assay was carried out as described in Figure 1E, except that one protein was incubated first with the probe for 30 min and then the second one added in 1X, 2X,10X and 20X excess (illustrated by the increasing triangle slope). Asterisk (*) indicates a non-specific band from the TnT. FP stands for Free probe.
(TIF)

Figure S8 **(A)** Expression levels by qPCR of *HY5, PIF4* and *PIF5* at T2 and T20 of a 17°C Red diurnal cycle in *hy5-215*, Col-0 and *pifQ* (*pif1-2 pif3-3 pif4-2 pif5-3*) backgrounds. Plants were grown and sampled as in Figure 4. **(B)** Expression levels for *PIF1* and *PIF3* under the same conditions as (A). Samples were normalized against ACT7 expression. Error bars represent ±SE of biological triplicates.
(TIF)

Figure S9 Expression levels of *PSY* and *GUN5* in Col-0 seedlings treated with or without an EOD-FR light treatment (T12) at 17°C. Samples were obtained and processed as described in Figure 7 and Figure 6. In brief, expression levels were measured for **(A)** *PSY* and **(B)** *GUN5* by qPCR on day 14^th^ in plants grown in 17°C Red diurnal cycles. Samples were treated with (+FR) or without (-FR) a saturating EOD-FR (T12) light pulse on day 14^th^ and harvested 3 h after treatment (T15). Levels are expressed relative to Col-0 (-FR) T15 sample. Error bars represent ±SE of biological triplicates.
(TIF)

Table S1 List of primers used for qPCR analyses, EMSA assays and ChIP tests.
(DOCX)
