# Supplementary figures and images for: The HY5-PIF Regulatory Module Coordinates Light and Temperature Control of Photosynthetic Gene Transcription
Source: PLoS Genet. 2014 Jun 12;10(6):e1004416. doi: 10.1371/journal.pgen.1004416 (PMC4055456; doi:10.1371/journal.pgen.1004416)

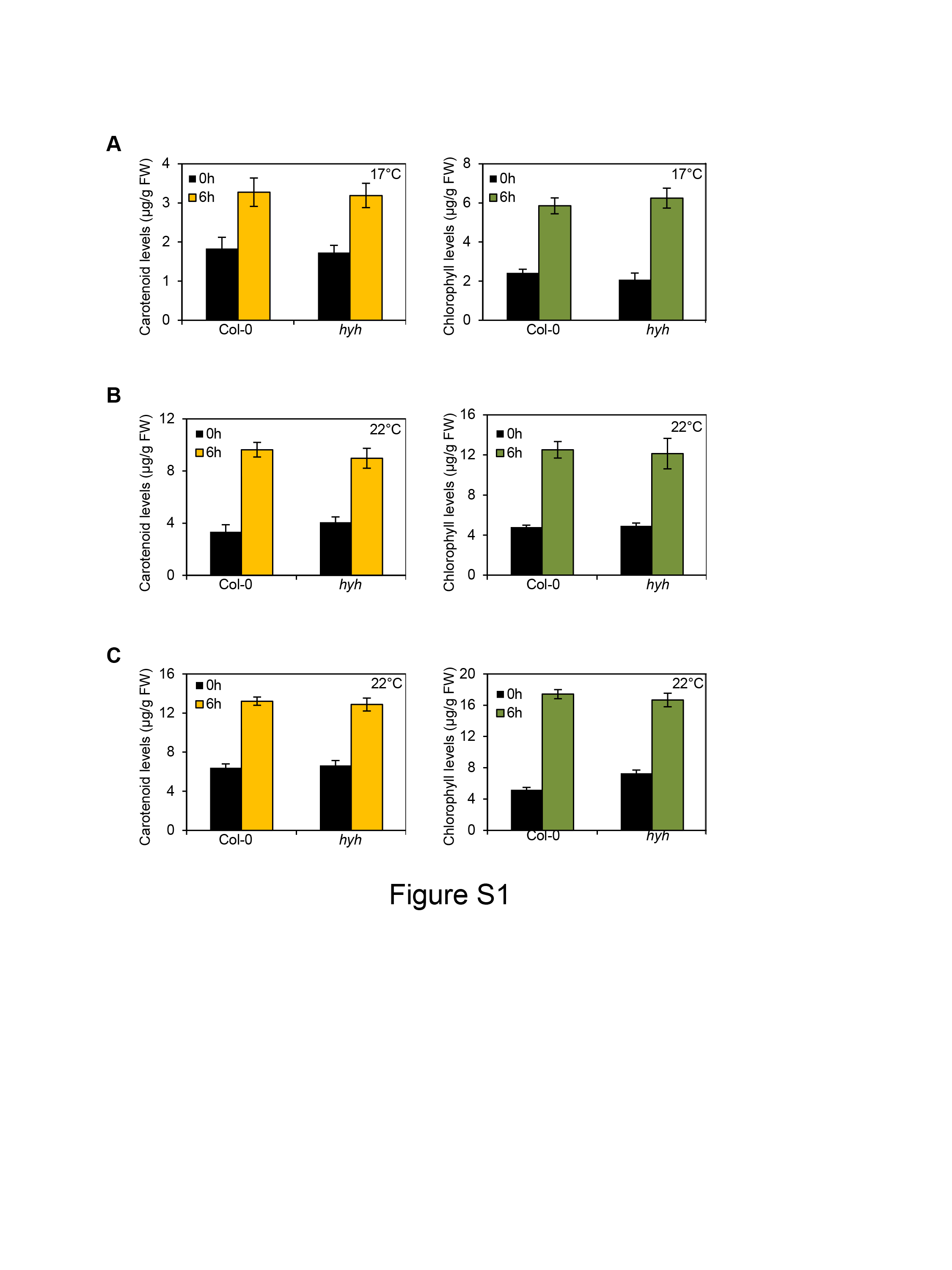

Supplement: Figure S1 — hyh does not affect photosynthetic pigment accumulation during Red controlled deetiolation. (A–C) Carotenoid and chlorophyll accumulation in Col-0 and hyh 3 day-old seedlings grown at different temperatures (17, 22 and 27°C respectively) in the dark (black columns) or after 6 h Red light illumination (40 µmol m−2 s−1) (yellow or green columns). For measurements seedlings were kept for two days at 22°C and 1 day at the indicated temperature in darkness. On day 3 they were subjected to red light treatment (for 6 h). The control set was kept in darkness (0 h time point). Graphs represent the results for biological triplicates sets. Error bars indicate ± SE. (TIF) [file pgen.1004416.s001.tif]

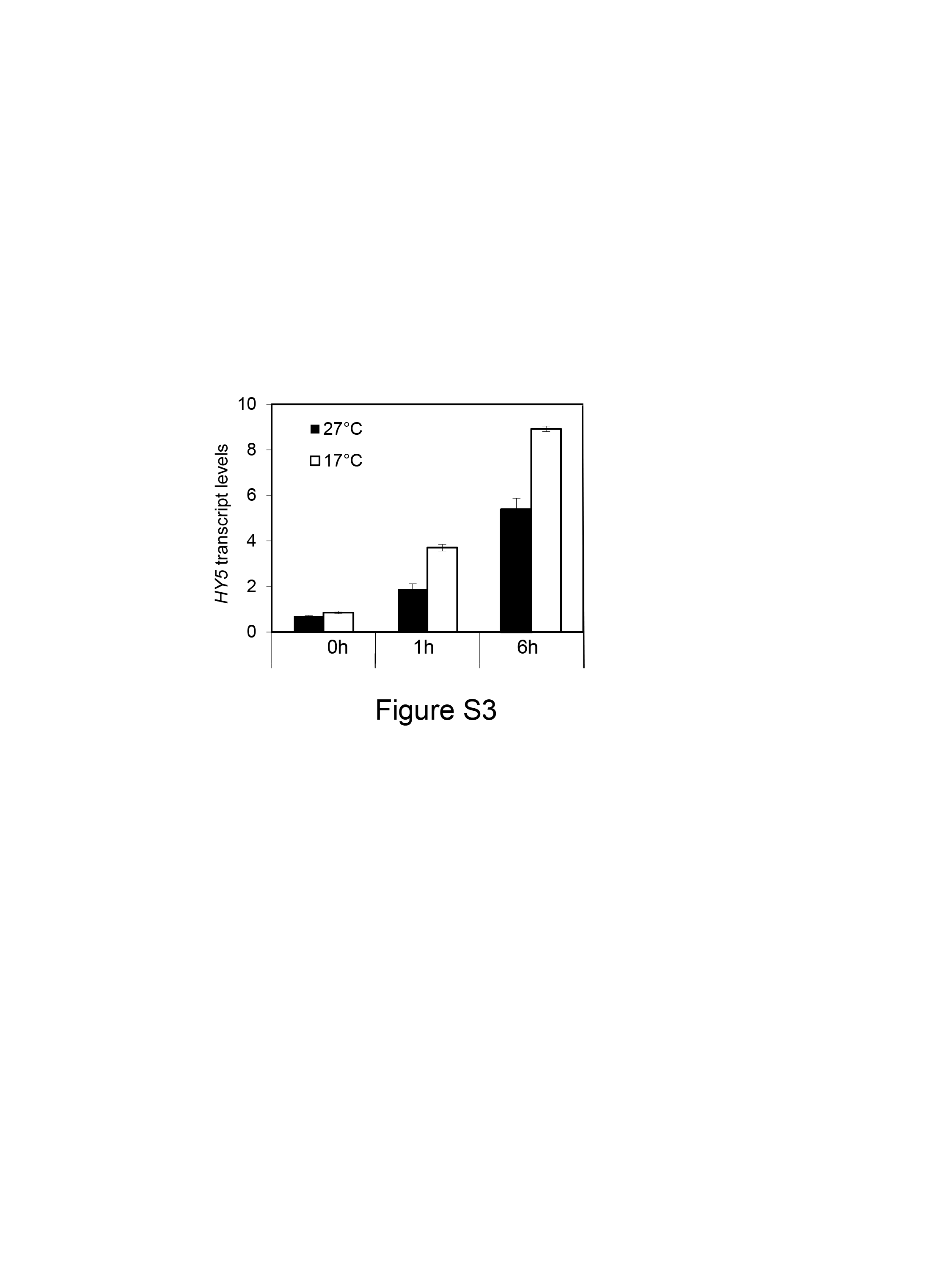

Supplement: Figure S3 — HY5 transcript levels at 17°C and 27°C. HY5 expression was measured in Col-0 seedlings grown in darkness for 5 days at 17°C and 27°C before Red light (40 µmol m−2 s1) illumination for 1 h and 6 h. Quantification of transcript levels was conducted by qPCR and expressed relative to ACT7 levels. Error bars represent ± SE of three biological repeats. (TIF) [file pgen.1004416.s003.tif]

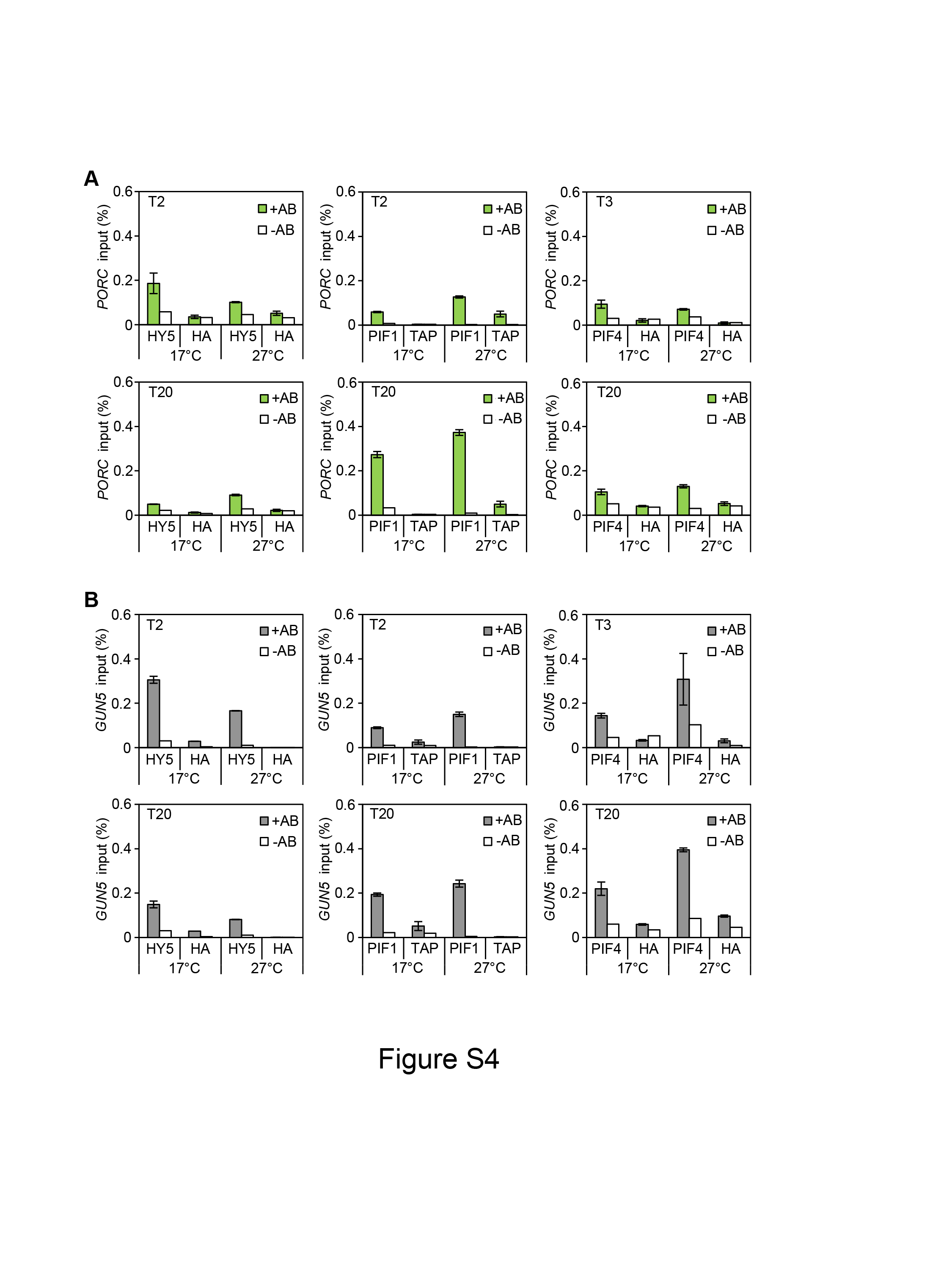

Supplement: Figure S4 — Chromatin immunoprecitation assays for PORC (A) and GUN5 (B) G-box regions in 35S::HA-HY5, 35S::TAP-PIF1 and 35S::PIF4-HA backgrounds. Plants were grown as indicated in Figure 4 under Red diurnals (12 h dark/12 h light) cycles at 17 and 27°C. Samples were taken at T2/T3 and T20 and processed in the same way as samples from Figure 4. Error bars represent ±SE of biological triplicates. (TIF) [file pgen.1004416.s004.tif]

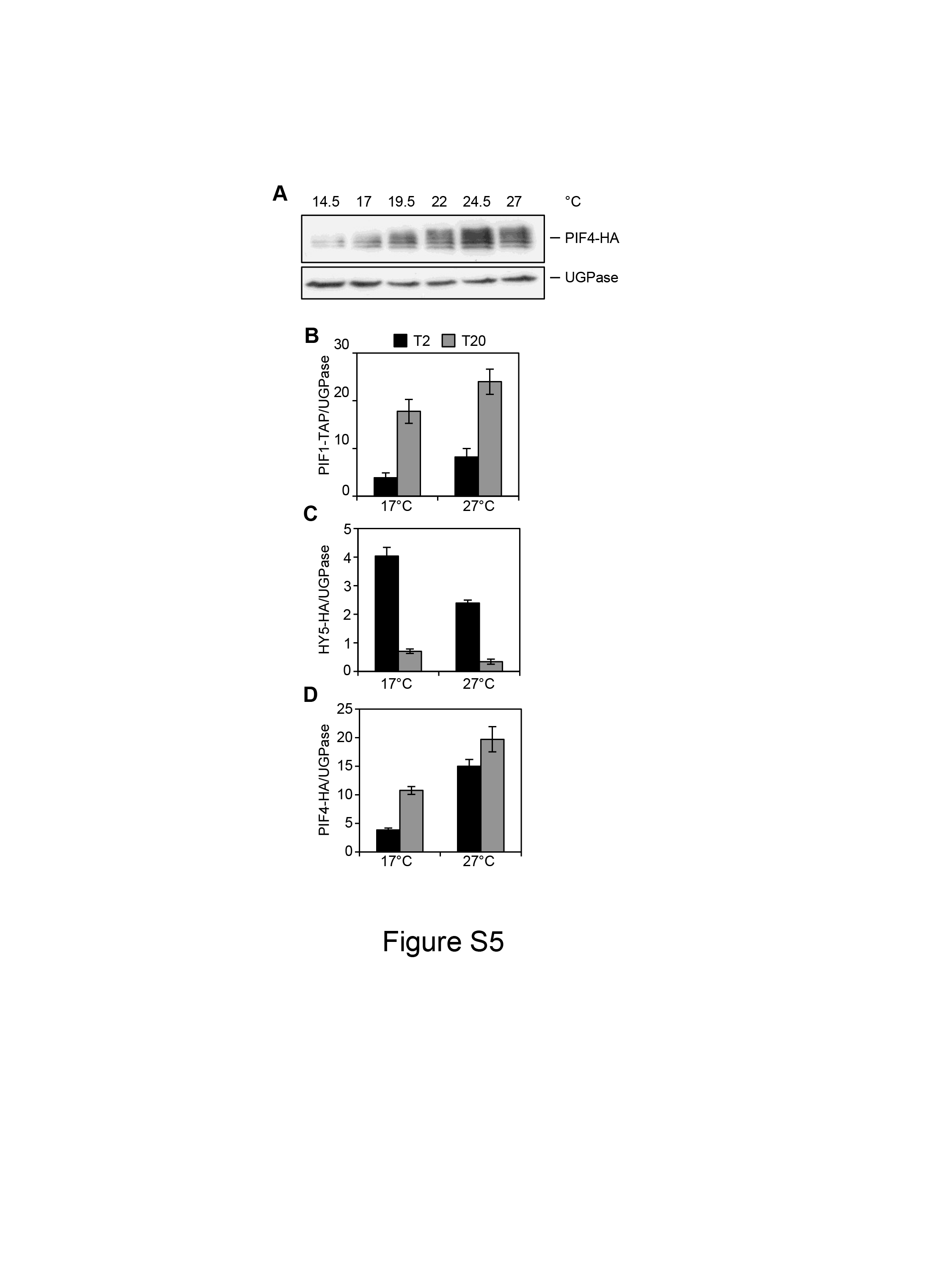

Supplement: Figure S5 — (A) Higher mobility PIF4 forms accumulate as temperature increases. Immunoblot of 35S::PIF4-HA protein extracted from 6 day old seedlings kept in the dark. Seedlings were grown at the indicated temperature. Immunoblots were carried out with an HA antibody. Loading control indicated by UGPase signal. (B–D) Quantification of protein abundance for samples grown in the same conditions as the ones used for ChIP at T2 (or T3 for 35S::PIF4-HA) and T20 time points in Figure 4. Protein was quantified by immunoblots for 35S::PIF1-TAP (B), 35S::HA-HY5 (C) and 35S::PIF4-HA (D) at 17 and 27°C during the morning (at T2/T3, black bars) and the evening (time T20, grey bars). Antibodies against the tag were used for signal detection (anti -Myc or anti-HA) and relative quantification was carried out against UGPase signal. Error bars represent ±SE of biological triplicate samples. (TIF) [file pgen.1004416.s005.tif]

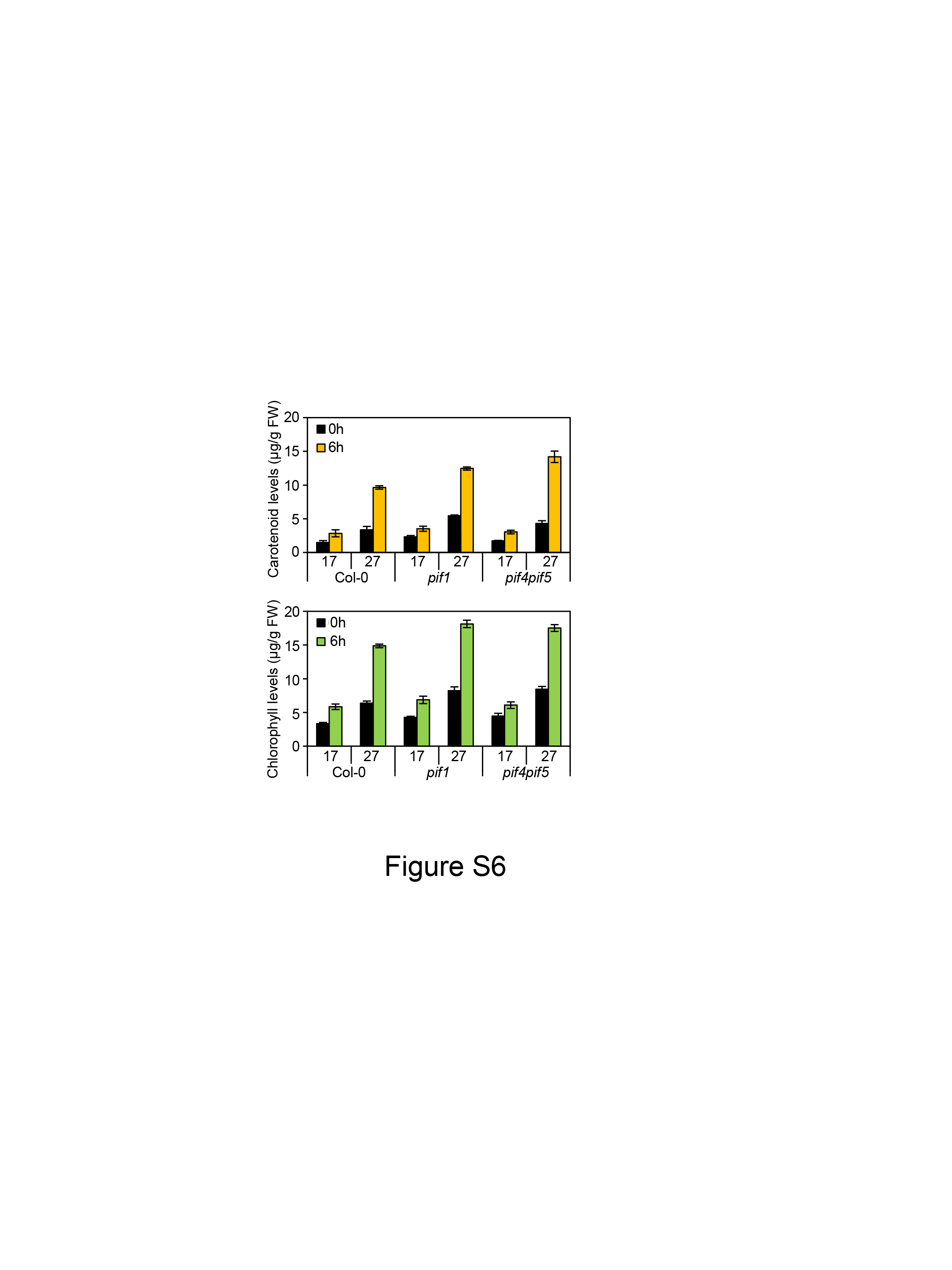

Supplement: Figure S6 — Carotenoid and chlorophyll accumulation for the pif4pif5 (pif4-2 pif5-3) double mutant compared to pif1(pif1-1) and Col-0 at 17 and 27°C. Plants were grown and processed for pigment extraction as indicated in Figure 2. Error bars represent ±SE of biological triplicate sets. (TIF) [file pgen.1004416.s006.tif]

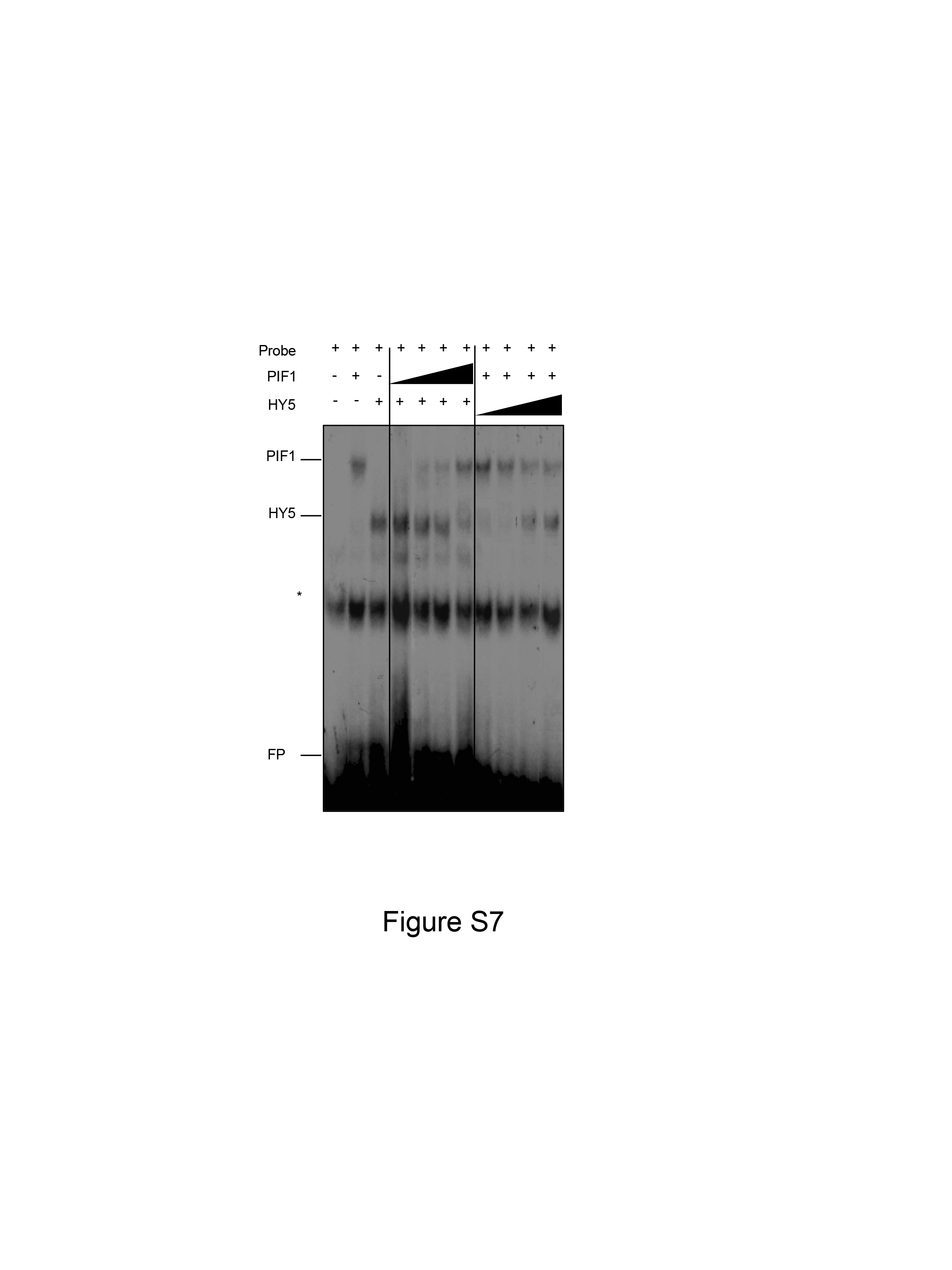

Supplement: Figure S7 — Simultaneous binding of PIF1 and HY5 to the PSY promoter in EMSA. The probe preparation and assay was carried out as described in Figure 1E, except that one protein was incubated first with the probe for 30 min and then the second one added in 1X, 2X,10X and 20X excess (illustrated by the increasing triangle slope). Asterisk (*) indicates a non-specific band from the TnT. FP stands for Free probe. (TIF) [file pgen.1004416.s007.tif]

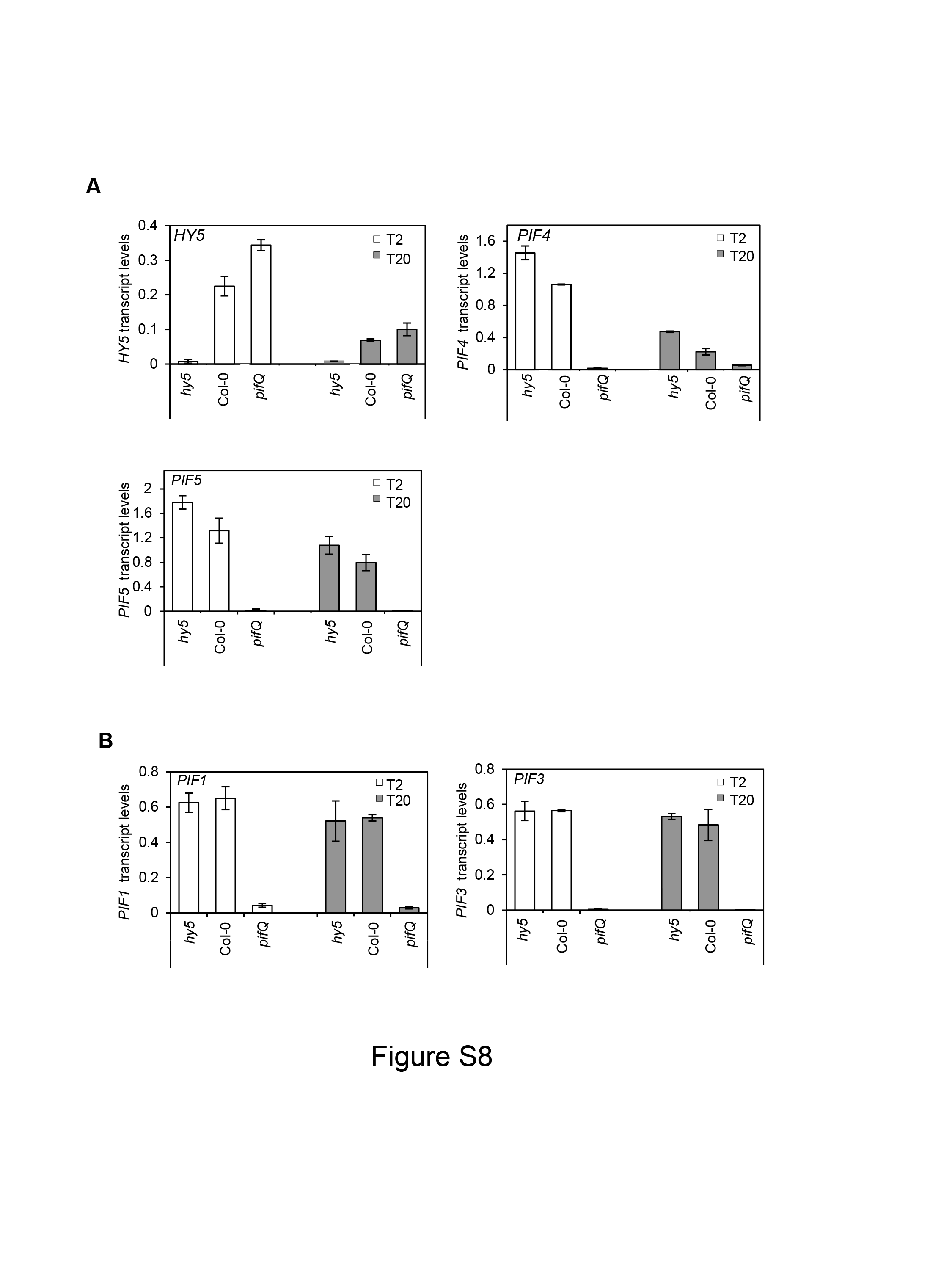

Supplement: Figure S8 — (A) Expression levels by qPCR of HY5, PIF4 and PIF5 at T2 and T20 of a 17°C Red diurnal cycle in hy5-215, Col-0 and pifQ (pif1-2 pif3-3 pif4-2 pif5-3) backgrounds. Plants were grown and sampled as in Figure 4. (B) Expression levels for PIF1 and PIF3 under the same conditions as (A). Samples were normalized against ACT7 expression. Error bars represent ±SE of biological triplicates. (TIF) [file pgen.1004416.s008.tif]

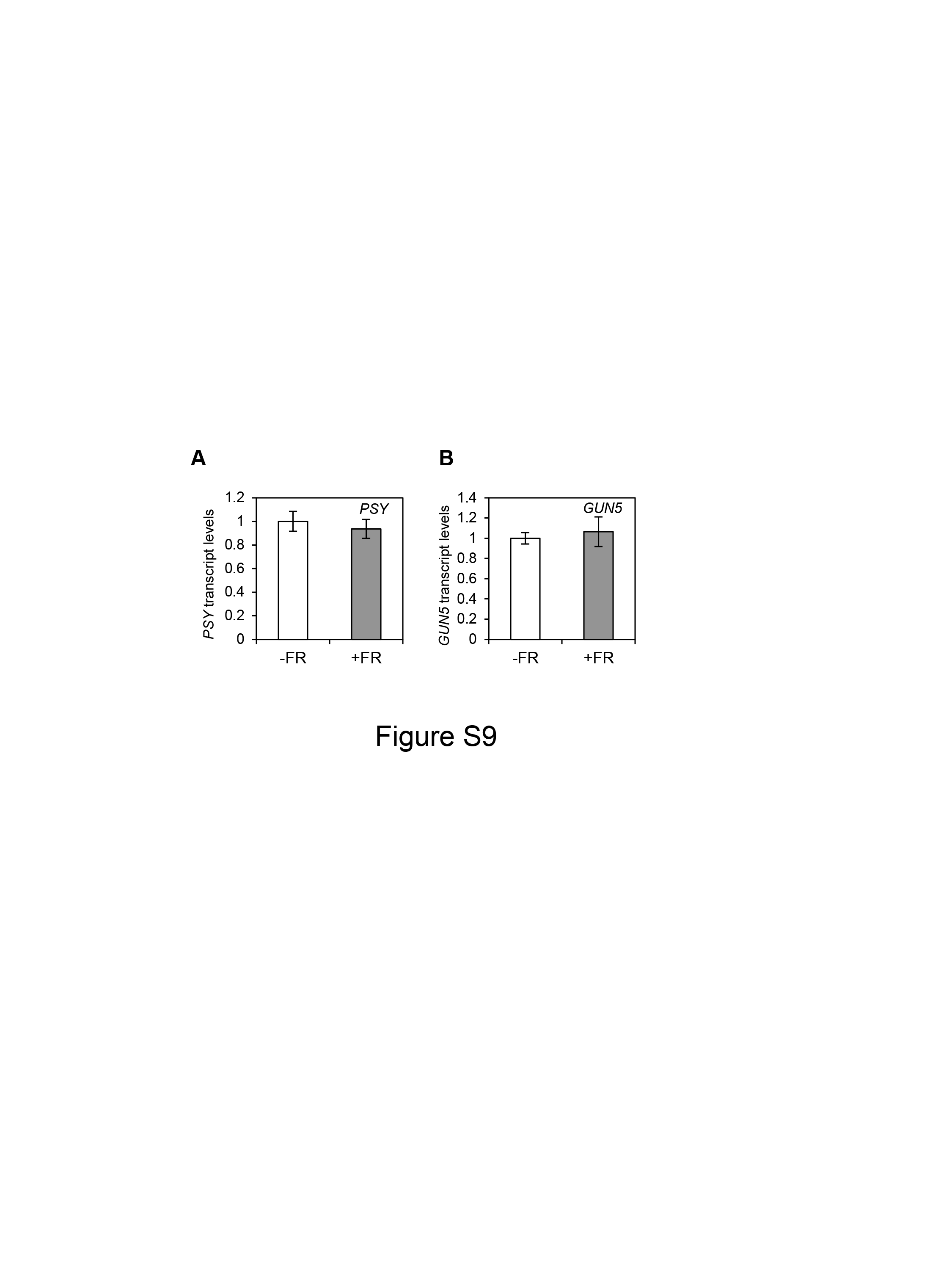

Supplement: Figure S9 — Expression levels of PSY and GUN5 in Col-0 seedlings treated with or without an EOD-FR light treatment (T12) at 17°C. Samples were obtained and processed as described in Figure 7 and Figure 6. In brief, expression levels were measured for (A) PSY and (B) GUN5 by qPCR on day 14th in plants grown in 17°C Red diurnal cycles. Samples were treated with (+FR) or without (-FR) a saturating EOD-FR (T12) light pulse on day 14th and harvested 3 h after treatment (T15). Levels are expressed relative to Col-0 (-FR) T15 sample. Error bars represent ±SE of biological triplicates. (TIF) [file pgen.1004416.s009.tif]
