# Supplementary material for: The HY5-PIF Regulatory Module Coordinates Light and Temperature Control of Photosynthetic Gene Transcription
Source: PLoS Genet. 2014 Jun 12;10(6):e1004416. doi: 10.1371/journal.pgen.1004416 (PMC4055456; doi:10.1371/journal.pgen.1004416)
Supplement: Figure S2 — Gene sequences and gene promoter regions under analysis. G-box motifs are highlighted in red. (PDF) [file pgen.1004416.s002.pdf]

Figure S2

Promoter and coding regions of selected genes.

ATG: transcription start site
TGA, TAA: transctiption stop site
Exon
Intron
G-box: CACGTG
Gene: At1g03630, *PORC*

cacaaagtccactaccagttcataatttccacaccatcatcatcatgttaccaaacagtgatgcatcatatagaacatacgttcattatcattcattt
tctatttatgtgttttggcttcattgattatcattttcatttcggtcataaattcaggttaatgtacaaaacataaaaatcgaaatcaactaagaaaat
aacaaaagaaaaaaaaatctattggaacaagagattgtaggcaggaagatcttgaatccgatgaatatcatccaaagaaagctctctctccagctgattc
cgagttgcttgtaatacttcctgataaacacacacagatatatacaattattaagaactaattctcaaaaaaaaaaaaaatgaaaagagaaagttgata
gattgagtgagaagagatacattgaattccatgtaagaagcgtgcattgcaagc cattgagcatccatcatcgcgaaagctatacagtatagtacatcga
atgccctctcgtcttctgttaatcattggaatatgattcttgattatttagcgatcgtcatactacactactaacttttagaaagaaggttttattcagt
ttaaaagaacatattcctattgtttaccttctaagagtttgaggaaattcattcctggaagacattttggctttgctgcacaaaaaacaaaacatgaatt
taaagccttttagaatcgaaatcgtatgtgaaaataacaagtatgacctgaataaaatggtgacagtttaagaacttacgattattttgtaaatcgagc
atttggatcaacatgaaggagatgttgattccggccactgcaaaagggtactcccactttgcccggtcgcctctttgttttaacaatagcctccggaaac
aaaccttcacaattttttatttgtataaacacattattttgtggagcaaagaagttcccaaa ccatgattattaaaaaaagtttaagtatatatgaacatg
tatttacgagtaatttaccggataagttcttgcagaaaatagtaaattttctagagcgataaatccgcaacccctacaaaacaaatgtttaacaaaacag
gcgtcaacactaagtattttttccttaagatctttttttgcaataagttaagtatagatcaaatcttacctgaaatcggtagatggattgggaccttgccat
cccatttctttccattgttctgttactaaacccgtgagatgaacattcggaaacgcgacgttccacaatgctttaagtgattcctgccattaatcacgca
aatatatttcacagaattcttatagctaaaacaatatatctgcatgttgtttgctcactgcgttaaacatcttactgataaagtgaattcacctacctga
tgatcaggacgttctcgtcgaaaggtacaaccatacgatcttgcagcctttgaagtctttcctcctatgtatgattgatttacacagatccaaaagtgt
tacaaactatttataatgtcttgatttcattttatgaagatatatgaaacttgtgattatgctaacctgaagaggagacaaagtgtagtcgactgccttg
tcttgacgtctattggagcgtgtgaagagccctcctatccatgatcttgttccaaccatacccgtagctgtaaccactggttcactaatagaaaattata
ttcatatatgagataatagatctaaattatgtatctaataaactaatcacatgcatatttctatggtgtgtcatttataccctatgatatatctagcaat
atatactcactaaaacaatatttagccaaatgagctattacgtgagcggaactacttgaccgctgccatcgcggctctttgttgcctttttctcgcctt
tcccacgcttcatcctcctctacctgcaaacgttttcagacatatgtttgcgtataacattagctgcagaacataa aaaaaaaaaaagatcaactctgaa
gatcagaaagtttgagtgagcttaattcacttacaagttacaaccaaaacgacactcaattacggtacattgaagattaaagaaacgggtggaatacaca
agaacaatgtatatgcaagattgcagtggtgtcgttgattactctcaatttgatgataagattcaaatttgatatgagtttggagaagaagaagatgacg
aagagatcattacttggcgatgagacgaacaagcaggaaagtattgcctacgcttcctgaacttcatgagatggacacaaatgttcaaagaatagaagat
aactaatattgatatcttttaaactaagagaacaaagatgtcaaaaccgacataaatgcatggaaagaaagaatttaggtataataatcctacatccaag
ttatctagtcatcaaaacagagattagttaatgattttgcaaaagctaagacttctctgtagagaattggacagaaagaaagaggaggaagatttttata
cagagctaagttgatcacagataagttttagtgttttatttggttgcgtacaggtcagagcttattaattttaaaaatagaaaaagcattgcgtgacta
ttttaacacgtttaactaaactagacagaattgtgggttgttctgtataaattttgtatttgtatttttatttactcctttgagttaatttagagaatga
caagaagatgatagaatcaaaatgactaaatataaatgagctaaagttctaattcgatttctaaatattcaaaagttccatcatcatttccattttcact
tagtcaaattttggtataattgggctcaaaaaggtttctctaaaagttgtagctaacataatagcgcaattaaaggatgttttcttttcataacttttat
gtaaagtttaaaactttacaatgaaattacccttgaaaatataattagcaaaca atgatgatcaaatcaaatttcacaagcaaaaatcgttatgattgat
taaaaagatgaggggggaaaaagttacctgttataggcatatgaacaagacaatggaggaggaggaggagatggtctagaaaccgtgacgacggaggagg
aaaagaaaagagatgaagtagaagaagaaaataaaataaaatagaggcaaaaatggaaattttcattggaattttgcaactttagaaattgtagaagat
gaagagagctgtgtgatccaaaagtgttttttttttctctctctttttttcgtttttgttttctttctcccacctctccattgaccgttaagttgccgcctt
cttctctcttccaccaattgttcttgacacatttggacggtccagattcattacagttcacctatcaacggtgatcagatcagttgagagttaacaaaa
caatattaaagcccatctcaaggcccaataaacgagagatgaagaatctgaaactagtgat ctgattggttgagggaaaagctgtagcctggaagacac
gtgtcactaacccaaaactaccttatcttctgtcgctcctcaattcggggaaaatcgaagacactgtgacactctagcaccttcagttccgtacaacaat
GGCTCTCCAAGCTGCCTATTCTCTTCTTCCTTCTACCATTTCAATCCAAAAAGAGgtatgttttcaccattagtaatgatttccatcttgttatgatttgttttcgataaaatgaagaatttacctcaat
gcaatggtgattttgcagGGCAAATTCAATGCTTCTCTAAAGGAGACGACTTTTACTGGTTCTTCATTTTCAAACCATCTTAGAGCTGAAAAGATATCCACCCTGTTGACCATC
AAGgtaaagtcaatcaccctatacgatgtgaaattctcaaaaaaagatatcgaaaa cgtcctgaggtttatactctgtttcgttgcagGAACAGAGAAGACAGAAACCGCGATTTTCCACCGGTATTC
GTGCACAGACAGTTACAGCCACGCCGCCGGCAAACGAAGCATCGCCGGAGCAAAAGAAGACTGAGAGAAAAGGCACTGCAGTGATCACTGGAGCTTCGTCTGGTTT
AGGTTTAGCTACGGCCAAAGCTTTAGCAGACACAGGGAAATGGCATGTGATCATGGCTTGCAGGAACTTTCTCAAGGCCGAGAAGGCAGCGAGATCTGTTGGAATG
TCCAAGGAAGATTACACAGTGATGCATCTTGATCTTGCTTCGCTTGAAAGCGTGAAGCAATTCGTTGAAAATTTCCGGAGAACAGAACAACCACTCGATGTTCTTGTCT
GCAATGCCGCGGTTTACCAACCAACTGCTAAAGAGCCTTCTTTTACAGCTGAAGGCTTTGAGATAAGCGTTGGAACCAACCATCTCGGTCACTTTCTTCTCTCAAGATTG
CTTCTTGATGACCTGAAAAAATCTGATTATCCATCAAAACGTATGATCATCGTAGGATCTATAACAGgtaaataacacagacaacttgtccatttccAccaagatagttcctagtatcaaac
aatgaatgttcttttctatgttcttgcagGAAACACAAATACTTTGGCTGGGAATGTACCGCCAAAGGCAAACCTTGGAGACCTAAGAGGCTTAGCGTCAGGATTGAATGGGCAA
AACAGTTCAATGATAGATGGAGGAGAGTTTGATGGAGCAAAGGCTTACAAAG ACAGCAAAGTATGCAATATGCTGACAATGCAGGAGCTTCACAGACGTTACCACGA
GGAAACAGGAGTCACGTTTGCTTCTCTTTACCCTGGTTGCATCGCTACAACAGGGTTGTTCAGAGAACACATACCGCTGTTTCGGCTTCTTTTTCCGCCTTTTCAAAAGTA
TATCACCAAAGGTTACGTATCTGAAGAGGAAGCTGGCAAAAGACTAGCACAGgtcagtttgcattgcaaatcctttctaatatatgtataacagattattgattgatactgaggatgtggttttgttt
tagGTTGTGAGTGACCCTAGTCTTGGGAAGTCAGGAGTGTATTGGAGTTGGAACAATAACTCGTCTTCGTTTGAGAATCAGCTTTCTAAAGAAGCAAGTG
ATGCGGAGAAGGCAAAGAAACTGTGGGAGGTTAGCGAGAAGCTTGTTGGTTTGGCATGA

ATG: transcription start site
TGA, TAA: transctiption stop site
Exon
Intron
G-box: CACGTG
Gene: At5g17230, *PSY*

agtagccaagctaattatcactatctctaaacgcttgtgattctagattgtacacgttataagtaccaggatctatggaattaagttgagtaaaaacaaa
gagtgatagtcactgactcaaacaaaaacgtcttggtcagtatatataacttactaaaaaaacaattaaacttgttataccatttgaaatttactactca
tcaatcatcatgtctgttaaattttcagtatattataaaaacgcatttccattaaatcaaagttaattatgccaattttcagtattctattgataactcg
ggctaatccacatcgaagctcgaccaagtcccatgcatatggtctaggtgatcctctagaactagggcggttgacataaatttcgtacgaaaacaatact
tttatcattcagtactaaaccaaaaaaagcggtttctttacgagagaatgtatttgaaacaagatacggagaaaatacggaccagactaaaaataacaaa
ggataaaaccaaaatcaagaagaaacttaaaagacaaaaccttctgagcgcaagagagagggccaagttgcgaagacagtatgggacccaaataccgact
aagcggaaatctaactttaacttttcctaccaatgaaacattgacacgtggaaaagcaacgtcgcccactttaccctccgcactttgaaatctttgata
agcttatcgctctcccattggccgtaggctccacgtggcatctctctgatctctcttctgctaatcctttttttacccaaactggcatgaccccacccta
aacccctattgtcctttctaccaacaattacttaatgacttatataatattctaattatat tttaattatgtaattaaataactattgatcccatctcca
tagtgacatgtctcacttttttcttccacaacaaaatgcgtcactcttgattcatcatgttcgtagcaaaatccacgttattacaaaaaacgttatacatt
ttgaaaggtaatccataaaatagtgagatggatcaagttttctacgcaaccctcgtagaattttagcaatgatccaaagctactgtatatatatagttac
gtttcgtaacacaacatgtattttaccatgattatgcctcttttttttttctttccacaacatatatgtatttacatccttatttggtttaggctgagtt
tgttaacatagcatcaccaatagggacaattttttttttttattacaatctaaatatctaaaatataaattcacatgaaattaatgtttctaatagagttc
ttcaatcttcatgtacaattgcacattttttgtgaaattattattactttatcattttttcttttaaaattaattcaccgagtaccgataaatgtttatatt
gaatacgatttagtaaacgacatatatgtttcgtcacattttttatcactatttgtaatatatctgttaaatttgatgatgtagtttctgtttttgaattg
aaaacttacaaagtttagaaaaaagaatgtaaattagaaataaatacactttct gtatttttgaatactataataggctaaaatcacatcattttctaat
gttgctttaaataatatatatcaaataaaggcttaaattacatttatataagaaatattgtaatacaaatatcaatttagagctattaatttacaattac
aacctgttgcccaattgatgaataaatcaaacatgtaattaaccaaactttctaattgatcaggaatcaatttagctaaataataagacataaatttggc
caagaaaaagaagagaaaacatgttaaataggtaatgggttttaatattgggatagttacctttatttgaaaactattaaggtttgatttaagacacgag
tcactcgagagtcgcatctcgccagtcaaaaactaaattaaaaaactatatagacccaaaaaataaaaataacgactttgtatttatactggcaagatt
ctcattaaaacccagcaaaaaaaaaaaaaaaaagacaaagaaggaagaaaatttgagtgggt gagaatttttttacgatagaggaagagacagcatcatcta
cttgtgttgtctgtgtacatatattacagtaagcgttgcaatataacttcttgaggatcttctcacattaatgggtcaaacc ttttgctcttccttttga
ttaatttagtgtttgacaatctcctcctccttctccttcttcttcaaagttttgtcgcagtatctattgttcttacagagagaaaggtaggcttttgtgt
ccatcactcattttcgctcgtttggttaagcttcatctgccatgtggttcactgttttgatacttttgggctcttatacctaatgttgatgtaacacaac
gatacataatctaatttgttttcgagtggaaaatagttgaacccagttgagtatttagccaatacgattagaggatttacttgtttttttctcttttcatg

ttcttcatttgcttttgaagatcttcctctttatgtttgtgtctcttcgctattttacttgcttgaagaaattgagtttctcatctcgcttaaatttggc
tttatatacagatttagagatctcgattcctaatctacttgtttttgtattcaatttgcaggaaagctttagtcttttaccagtttgatccaattctggg
tttcactgaaaaaaagttgggagtttgattcttctaactgtagaagaaacagagtcaacagaagaaaactaaaaaagttgagattttctctcacgcgct
caagaacttgagtATGTCTTCTTCTGTAGCAGTGTTATGGGTTGCTACTTCTTCTCTAAATCCAGACCCAATGAACAATTGTGGGTTGGTAAGGGTTCTA
GAATCTTCTAGACTGTTCTCTCCTTGTCAGAATCAGAGACTAAACAAAGGTAAGAAGAAGCAGATACCAACTTGGAGTTCTTCTTTTGTAAGGAACCGAA
GTAGAAGAATTGGTGTTGTGTCTTCAAGCTTAGTAGCAAGTCCTTCTGGAGAGATAGCTCTTTCATCTGAAGAGAAGGTTTACAATGTTGTGTTGAAACA
AGCTGCTTTGGTGAACAAACAGCTAAGGTCTTCTTCTTATGACCTTGATGTGAAGAAACCACAAGATGTTGTTCTTCCTGGGAGTTTGAGTTTGTTGGGT
GAAGCTTATGATCGATGCGGTGAAGTTTGCGCTGAATATGCTAAGACGTTTTATCTTGgtatggatctttttaactcttctttggctttggtgtaatgta
GagtggtgttctgagttctaatgtttgtgttgcagGAACTTTGCTTATGACACCCGAAAGGCGAAAGGCGATTTGGGCAATCTACGgtaagttactgcac
Aaaaccaatggttgaagagctgtttagaatatttgcattgtcaacattgattataattgtaggataaag TGATGCTCAAAGTAGATTTCTACAAACAAT
CTATTGTGGCTCTTGgttagtatctagttaagttgtcttaacaagagaagttgaagctgaaatagattccttgaatcttacactcatgtagaaagatagg
tctctagttagtttgtgtggttaccttgtgtctaagataagtccatgaacaccaagcatccaaatttgcaccctttgaagataaagaataaagacccaaa
aagactagcttagttactaatatgggtttgtccaaaaaaaaaaaagttactaatatgggtttttaatgggctttttggcccaatggtcttaaactctgg
agattttactctaatgacccctgtctaggacatactagtatccaaagcatatggggaattttaaagttgttttcttatatgtttgattggtttgaattt
gatagttttactctgtcttttgtgcagTTTGGTGTAGAAGAACTGATGAACTTGTGGATGGGCCAAATGCTTCACATATAACTCCCATGGCTTTAGATAG
ATGGGAAGCAAGGTTAGAAGATCTTTTCCGTGGTCGTCCTTTCGATATGCTTGATGCTGCTCTCGCTGATACAGTTGCTAGATACCCGGTCGATATTCAG
gtcagccttgctctgtcttatcttacttcctcatttcattacatgagttaagagttgcattcagtccattgcaataaaccttataaactttggtctaat
gtttatttatgttgcagCCATTTCGAGACATGATCGAAGGAATGAGAATGGACTTGAAGAAATCGAGATACCAGAACTTCGATGATCTATACCTTTACTG
CTACTACGTCGCTGGAACCGTCGGATTGATGAGCGTTCCGGTTATGGGAATCGATCCTAAGTCGAAAGCAACAACCGAAAGTGTTTACAACGCTGCCTTG
GCCCTTGGTATAGCCAATCAGCTTACTAACATACTCAGAGACGTAGGCGAAGAgtgagtcttttttatatattttaaatagagctttggtttcttgatttc
acatatctcacttaaaccggtttcttgatttcacatgcttcttttaaaccggtttcttgattcacatatctctcttaaattggttttgttctcactatca
gTGCGAGAAGAGGAAGGGTTTATCTGCCTCAGGATGAATTGGCTCAGGCTGGTCTTTCAGATGAAGACATATTCGCCGGAAAAGTAACTGATAAATGGAG
AAACTTCATGAAAATGCAGCTTAAACGAGCAAGAATGTTCTTCGACGAAGCTGAGAAAGGCGTCACCGAGCTCAGTGCCGCTAGCAGATGGCCT gtaagt
Gtctctaaaacactcttcagccgcaagtttaccgtttacttatttcttttggatcgattttaaccccgaggacttggctgcaatgtttcag GTATGGGCT
TCATTGCTATTGTACAGGAGAATACTGGACGAGATTGAAGCGAATGATTACAACAATTTTACTAAGAGAGCTTATGTGGGGAAAGTCAAGAAAATTGCAG
CTTTGCCATTGGCTTATGCTAAATCAGTACTAAAGACTTCAAGTTCAAGACTATCGATATGA

ATG: transcription start site
TGA, TAA: transctiption stop site
Exon
Intron
G-box: CACGTG
Gene: At1g08550, *VDE1*,*AVDE1*, *NPQ1*

actattgttcccacacacaaaaattattcttagtatttggtagctgtagctgttgtaatttgaagtccacttgataacttccttgtatttgtaggcgaag
tatttagaagctttagcctctgagacacttctcacaagcgatgaagcagtagttgtagcagcagcagctgaagcagtcgcccttgcaagagctgctgtca
aagttgccaaagatgcaacattatttaagaacagtaacaacacgaacctattaacttcgtcaacggccgacaaacgctccaagtgggaccagtttactga
gaaggaacgtgctggcatattggggcatctagcggtttcggacaatggaattgtgagtgataaaatcactgcatctgcctctaacaaagagtctattggt
gatttagaatcagaaaaacaagaagaagttgagcttctggaggagcaaccttcagtgagtttagctgtgagatctacacgtgaaactgaaaggaaagctc
ggagggcaaaagggttagagaaaactgcatcaggtattccgtctgtgaagactggttcgagccctaaaaagaaacgtcttgttgcgcaggaagttgatca
taatgatcctttgcgttatctaagaatgacaacaagcagttccaagcttctcactgtcagagaagaacatgagctgtcggcaggaatacaggtttgtcac
caatattccaaattctaactaaccacctccctttttctacatcgcgtctgagacatcacaaacactcacccttcgtaggactctagattctaggcgaaa
atgatgtctacaaaatctttagatgtttgagaatgacgaaattagtggttagtggtagaatccaagatcgcttctttgtgctttctagccaatatatcat
ctgaagttctgtgaaagctaattatcctgacatgcttgtcttaaattgaattgtcaccttttttgttcttattatgtagccattcaccaactgtttagtg
ttgatagaaaatgggaaacgtatgttcagctttggaagaatcaacattacttgtgaacgttatttcttcttctctgtaatttttcttatctttctttatt
gtggttttgatcacacactttggtttctttgtttaggaccttctgaagttagaaagacttcaaacagagcttacagagcgtagtggacgtcagccaacct
ttgcgcagtgggcttctgctgctggagtcgatcagaaatcattaaggcaacgtatacatcatggcacactatgcaaagacaaaatgatcaaaagcaacat
tcgactcgttatttcgattgcaaagaattatcaaggagctgggatgaacctccaagatcttgtccaggtttgagcttttctctccttattttttttgta
ataacacatctgtaagcagtagtgatgaaagtgatagatattgaacaccttgctcaaatattttcgaaatggtcacaggaagggtgcagagggcttgtga
ggggagcagagaagtttgatgctacaaagggttttaaattttcgacttacgcgcattggtggatcaagcaagctgtgcggaagtctctctctgatcagtc
cagaatgataagattgcctgtaagttatcccaaatgtttttctttttttacttgtttaaacaaaaggtttgggattatgcctcaaattttaaggatgatt
ggcagtttcacatggtggaagcaacatatagggtgaaagaggcacgaaagcaactgtacagtgaaaccggtaagcacccaaagaacgaagaaattgcaga
ggcaacagggctgtcgatgaagagactcatggcggttctactctctcctaaacctccgaggtcgctagaccagaaaatcggaatgaatcaaaacctcaaa
ccttcggttggtaccacctgacacaacacacaccatacaacatagacttgttgtttgtctaaaaatagtaaataatatgagatgggtttgtaatgtaaaa
atatgcaggaagtgatagcagatccagaagcagtaacgtcagaagatatactgataaaggaattcatgaggcaggacttggacaaagtgttggactcgtt
gggtacaagggagaaacaagtgatacgttggagatttgggatggaggatgggagaatgaagacgttgcaagagataggagagatgatgggagtgagcagg
gagagagtaagacagatagagtcatctgcattcaggaaactaaagaacaagaagagaaacaaccatttgcagcaatacttggttgcacaatcataattct
ttttctcttcaatatggaatgttttgtgcatagaacccacccacctgagcataacaagcagtaaattattactttttagcatttacgattcttgtgacaa
ctactaaatcgaaaaaattattttaaaactaaaaagaaattgttgtctgaaaaacataagtgatatacaacaataaataaatatctcttataagaatttg
ggaaaaaaaaaatccaaagattagagatatttgggtggagaaaacaaccgcctaggacaaaaaaaaattgtgggtagaggaggaggaggaggctttgttc
acacaccacacgacggaacattctcttccgagtcttatccaaagcaaaatcatattttgcttaaatatcttcttctccggctctgtctctgtctctcttc
ttcccgcggagctttcagttttgtttggtaacatcgtttctctttctcactaactctgatatcatattatcaattttttcctttcaacacccaaaatca
caatctttcagctttattctgtttttccttgcttgagaattgtgttcgattcatgtctacgaactgaaatcacaaatttgccaacgttttatgattcct
ggttccgtatttttttttttgtaatcaatagttagctcatttcgaataatcaaactcaatttttattgttcatgatttgttatctgggataaaagagagta
atgtgctaattatgtatactcaggtattgcttggtgtggggaagattagatagtgtgaagaATGGCAGTAGCTACACATTGTTTCACTTCACCTTGTCAT
GACCGTATTCGATTTTTCTCAAGTGATGATGGTATTGGTAGGCTTGGCATTACAAGAAAGAGGATCAATGGCACTTTCTTGCTCAAGATTTTACCTCCAA
TCCAAAGTGCTGATCTCAGAACAACTGGTGGGAGATCCTCACGTCCTTTATCTGCATTCAGGTCAGGATTCTCTAAGgtaatcaatcatactagtcttgt
GtttcaatacaaaatttggtatttcgatgatctcattgcttttgatcatgtcagGGGATATTTGACATTGTGCCATTACCATCAAAGAATGAGCTGAAAG
AGCTGACCGCTCCGCTGTTGCTAAAACTCGTGGGTGTTTTAGCTTGCGCGTTCCTTATTGTTCCATCTGCAGATGCAGTTGATGCACTTAAAACTTGTGC
ATGCTTATTGAAGGGATGCAGgtaactttccttcgtttggattctgcatatatagtgtcccccccaccaaaaccggtttattttcattgtgattgtgatg
ctataagtgttttgaattgtatagctccatactcttcaattgttgagaatgttaaagctagatgaagttactgtgtctgtctatgatagagctgaaatca
gttgatgatatctttctgatttttttgccgtcaatttatatatttccctccttgtctgaccatatggaagcccaattatctgttagGATAGAACTCGCAA
AGTGCATTGCCAACCCTGCCTGTGCAGCCAATGTCGCGTGCCTTCAGACCTGCAATAACCGTCCAGATGAAACCGAGTGCCAGgttataatttattttga
Tatatcattttatgaggcagagatattgcttgcttatgaaagaagaccaatgatttttgtttataattatgtgcttatattatatcag ATTAAATGTGGGG
ATCTGTTTGAGAACAGTGTTGTTGATGAGTTCAACGAGTGTGCTGTGTCGAGAAAAAAGTGTGTTCCTAGAAAATCTGATCTCGGAGAATTTCCTGCCCC
AGACCCTTCTGTTCTTGTACAGAACTTCAACATCTCGGACTTTAACGGGAAGTGGTACATTACAAGTGGCTTGAATCCAACCTTTGATGCCTTCGACTGC
CAGCTGCATGAGTTCCACACAGAAGGTGACAACAAGCTTGTTGGAAACATCTCTTGGAGAATAAAGACCCTAGACAGTGGATTCTTTACTAGGTCAGCCG
TACAAAAATTCGTGCAAGATCCTAACCAACCTGGTGTTCTCTACAATCATGACAACGAGTACCTTCACTATCAAGATGACTGgtaataacatcttgtatc
CacccacgagggagagtcttgatagtgctgctattcttctttggcctaacaagctttcatatgattttgcagGTATATCCTGTCATCAAAGATAGAGAA
TAAACCTGAAGACTATATATTTGTATACTACCGTGGGCGAAACGATGCTTGGGATGGATATGGTGGTGCAGTTGTATACACGAGAAGTTCTGTATTACCC
AATAGCATTATACCAGAACTCGAAAAAGCAGCAAAAAGCATAGGCAGAGACTTCAGCACATTCATTAGAACGGATAACACATGTGGTCCTGAACCTGCGC
TCGTGGAGAGAATTGAGAAGACAGTGGAAGAAGGTGAAAGGATAATCGTAAAAGAGGTTGAAGAGATAGAAGAAGAGGTAGAGAAGGAAGTGGAGAAGGT
CGGTAGGACTGAGATGACCTTGTTCCAGAGATTGGCTGAAGGATTTAATGAACTGAAGCAAGACGAGGAGAATTTCGTGAGAGAGTTAAGTAAAGAAGAG
ATGGAGTTTTTGGATGAGATCAAAATGGAAGCAAGTGAGGTTGAAAAATTGTTTGGGAAAGCTTTGCCAATCAGGAAGGTCAGGTAG

ATG: transcription start site
TGA, TAA: transctiption stop site
Exon
Intron
G-box: CACGTG
Gene: At5g13630 *GUN5*

aacatatttaagccttaagtaatgaagaaagaaacgtaacatggaacagaataatagaaagaacgatgagaaaagaacctctgtgttctgaaacgaaagc
cgccagtcatatgcagccatgtacatatttttctcttcatatccaatatgtgcaaggttagcaatcagcactgcccagacaaagtagccaggagcaaagt
agtcagcagccacgagtcctgatacagctcgaactctaataccagctggatccaacccagtttcattgtcaagtgacatgtgttccacccaacatagagg
cctgaatcaaagagataattgattagttaagatactatcaactataccactgttaccatcaacaataacattgcccatctctaccatagcaaaaacatga
acactgagccgaaagaagaagatgcagatcgtaacaaagatgcaacccacaaaacacttttttttttaatatatccatagcagggtcgggaggagttcaaa
gctggataacagaacgaggatggacgaaattcaagacatgtgaataaactcgatgtgaaactttgaaattgagaaaaaaattgagaaaagcacattgagc
tgaaagatcaaaaaggaactagctttaattacacatatacatagttgaataataaagcaacagtcctctatcacagcctgaacacactttccacagatgt
agttagaacttctctattcccaacaaaacataaaacactacttcattatacgctacacaaagctaaatctacacgcaacgacagtaaaggaagtaacttg
ttatgaacggactctagggcatgtctagcaacagatttggcaataagatgacttgaaattccaaggcagctattttatatcctaaagttaacaatcaaac
agaaatagtccaagccaattacgtccttggaacgcttaccttacacatacagaaatagtctcttaagaaaatagcgtttcacatcaaaaaaaaaaaaag

taaagagtaagacagacaaacatggaattaaagtttgagtgatgttgaagaagcaacaagtgcgtgatctcatcagatccaatccaaatcccaatataaa
ggaagagtgagaattgttgagctcaccttttgtagacttcaccaaaagttccaccccacaaacgttttctaaataaaccatcagcgcattgtttgccttc
ccaaagctcgagcccaccggtgacaatcccaggaatgaagacaacaggatgtttcgccttaagaccttctttttgagcttaacaccgggcgggtcaggc
aaaggacccgtgattcgctccgttacatactgagggaagctcgcaggcattgcgttgtaaaggaagagaagaaaccaccaggttacacacacacacccaa
tgaaccaacaacaagaatcgatgcacgaccacttccctcctccgttcgatttct tatggtgggatttggaagattcgtgtggtttcttttgcgaatcctc
atcgtgcaccacctcttcagatggcggcgtcgatggtttctccgtcggctttttccgatgaataaggggcatatctgtcacttgaaattaaaccaggtta
agctatacggactttgtcaaatctcgtccaggaatttagaacacagattagggattgttagatcagtcgagagagacaaagagctgtttctctgcgaatt
tctggaaccaatcaaatcaaatttgtctcttttgtttattgtcgacttttctgtttttttctgttctggtattattcctccaagggactcggaccggatg
gcaaaacttgaccactttgctcctggttcaggttgcggttcatggtcaaccttagacccgacctatccgtttcgttgatttcattaattagtctgcgttt
tcttgggtggagtgcagacagattttcttctgcagtaggcgttaatccgataggctcttgt tgggcctcaaatagttcagtaaacttttcgtggggcttt
tagtatagcccatgtattaataacaatattacacttgggaaatgacaaaaaaaaaaaagagaatgaagcggtactcttatatcaaaaaaacatattgtat
atcttcgaatatgatgcaactcatgcaacaacaatactagtaggctacatgtattgctccatttaattacatataacttaatattctaattcataaactt
tcttttaattatatattttatgtaagttttaaatattgaaaaaaaaaaaagttttaaatattgaaacatgataagtttttacatccatttattataaaaa
aaatagtgatgatgatgatcaaaattttgagtacattttagagtgttttaacttttacatttgttgttacaattgttatgagtctgatatttcaaatttc
gttttcaaagaaaaatcctgagtttaccttaaaagattaaagccagtttttaagaaataagtagaaaaatatcaaaccagacctagctgaaccaaaaatt
atacaaaatctagaccggattaaaccggctacccgacgaatatgcctagtaaaagtgtcgtcatcggaaaagaatcttttacatggctgaaaccgcaaat
acgatcttcaagagagcaacataaaaatacttgtcaaataagtaaagaaaataagacactcaatatccacactcccaaaccatgaaaatatgaatagaaa
tctcaatgagccaatcagaggccagcaaagtccatctcattgtcccaagcggatatacatccgacaaaactcaacctcacaagtagactctcccttcttc
cgtaactattcgtccacgtgtccttccctctcaccacttaccttcaaaccaacgcttctttttttagttccttggtccgaagcgtttaccgatgagagaa
tcataaactcccacttggagctcaaaaagtgtaagagacaaccaacaaaaaacgattcatctcttctcctatcct ctcctcttcgaattcaacgtttgga
gaatccagcagccgcaaaATGGCTTCGCTTGTGTATTCTCCATTCACTCTATCCACTTCTAAAGCAGAGCATCTCTCTTCGCTCACTAACAGTACCAAAC
ATTCTTTCCTCCGGAAGAAACACAGATCAACCAAACCAGCCAAATCTTTCTTCAAGGTGAAATCTGCTGTATCTGGAAACGGCCTCTTCACACAGACGAA
CCCGGAGGTCCGTCGTATAGTTCCGATCAAGAGAGACAACGTTCCGACGGTGAAAATCGTCTACGTCGTCCTCGAGGCTCAGTACCAGTCTTCTCTCAGT
GAAGCCGTGCAATCTCTCAACAAGACTTCGAGATTCGCATCCTACGAAGTGGTTGGATACTTGGTCGAGGAGCTTAGAGACAAGAACACTTACAACAACT
TCTGCGAAGACCTTAAAGACGCCAACATCTTCATTGGTTCTCTGATCTTCGTCGAGGAATTGGCGATTAAAGTTAAGGATGCGGTGGAGAAGGAGAGAGA
CAGGATGGACGCAGTTCTTGTCTTCCCTTCAATGCCTGAGGTAATGAGACTGAACAAGCTTGGATCTTTTAGTATGTCTCAATTGGGTCAGTCAAAGTCT
CCGTTTTTCCAACTCTTCAAGAGGAAGAAACAAGGCTCTGCTGGTTTTGCCGATAGTATGTTGAAGCTTGTTAGGACTTTGCCTAAGGTTTTGAAGTACT
TACCTAGTGACAAGGCTCAAGATGCTCGTCTCTACATCTTGAGTTTACAGTTTTGGCTTGGAGGCTCTCCTGATAATCTTCAGAATTTTGTTAAGATGAT
TTCTGGATCTTATGTTCCGGCTTTGAAAGGTGTCAAAATCGAGTATTCGGATCCGGTTTTGTTCTTGGATACTGGAATTTGGCATCCACTTGCTCCAACC
ATGTACGATGATGTGAAGGAGTACTGGAACTGGTATGACACTAGAAGGGACACC AATGACTCACTCAAGAGGAAAGATGCAACGGTTGTCGGTTTAGTCT
TGCAGAGGAGTCACATTGTGACTGGTGATGATAGTCACTATGTGGCTGTTATCATGGAGCTTGAGGCTAGAGGTGCTAAGGTCGTTCCTATATTCGCAGG
AGGGTTGGATTTCTCTGGTCCAGTAGAGAAATATTTCGTAGACCCGGTGTCGAAACAGCCCATCGTAAACTCTGCTGTCTCCTTGACTGGTTTTGCTCTT
GTTGGTGGACCTGCAAGGCAGGATCATCCCAGGGCTATCGAAGCCCTGAAAAAGCTCGATGTTCCTTACCTTGTGGCAGTACCACTGGTGTTCCAGACGA
CAGAGGAATGGCTAAACAGCACACTTGGTCTGCATCCCATCCAGGTGGCTCTGCAGGTTGCCCTCCCTGAGCTTGATGGAGCGATGGAGCCAATCGTTTT
CGCTGGTCGTGACCCTAGAACAG gttagtccacttatgaccatattgcatttttcatgattgtcattacgcaagagtgggaatgttttcttatggaggt
gtttaccaattgctgcagGGAAGTCACATGCTCTCCACAAGAGAGTGGAGCAACTCTGCATCAGAGCGATTCGATGGGGTGAGCTCAAAAGAAAAACTAA
Ggtattgtattaccactcatccttagtgaaatcacattccttcatgactttctcaagaatgtaatgtatgttctcatgaatcaactctggctcattgcag
GCAGAGAAGAAGCTGGCAATCACTGTTTTCAGTTTCCCACCTGATAAAGGTAATGTAGGGACTGCAGCTTACCTCAATGTGTTTGCTTCCATCTTCTCGG
TGTTAAGAGACCTCAAGAGAGATGGCTACAATGTTGAAGGCCTTCCTGAGAATGCAGAGACTCTTATTGAAGAAATCATTCATGACAAGGAGGCTCAGTT
CAGCAGCCCTAACCTCAATGTAGCTTACAAAATGGGAGTCCGTGAGTACCAAGA CCTCACTCCTTATGCAAATGCCCTGGAAGAAAACTGGGGGAAACCT
CCGGGGAACCTTAACTCAGATGGAGAGAACCTTCTTGTCTATGGAAAAGCGTACGGTAATGTTTTCATCGGAGTGCAACCAACATTTGGGTATGAAGGTG
ATCCCATGAGGCTGCTTTTCTCCAAGTCAGCAAGTCCTCATCACGGTTTTGCTGCTTACTACTCTTATGTAGAAAAGATCTTCAAAGCTGATGCTGTTCT
TCATTTTGGAACACATGGTTCTCTCGAGTTTATGCCCGGGAAGCAAGTGGGAATGAGTGATGCTTGTTTTCCCGACAGTCTTATCGGGAACATTCCCAAT
GTCTACTATTATGCAGCTAACAATCCCTCTGAAGCTACCATTGCAAAGAGGAGAAGTTATGCCAACACCATCAGTTATTTGACTCCTCCAGCTGAGAATG
CTGGTCTATACAAAGGGCTGAAGCAGTTGAGTGAGCTGATATCGTCCTATCAGTCTCTGAA GGACACGGGGAGAGGTCCACAGATCGTCAGTTCCATCAT
CAGCACAGCTAAGCAATGTAATCTTGATAAGGATGTGGATCTTCCAGATGAAGGCTTGGAGTTGTCACCTAAAGACAGAGATTCTGTGGTTGGGAAAGTT
TATTCCAAGATTATGGAGATTGAATCAAGGCTTTTGCCGTGCGGGCTTCACGTCATTGGAGAGCCTCCATCCGCCATGGAAGCTGTGGCCACACTGGTCA
ACATTGCTGCTCTAGATCGTCCGGAGGATGAGATTTCAGCTCTTCCTTCTATATTAGCTGAGTGTGTTGGAAGGGAGATAGAGGATGTTTACAGAGGAAG
CGACAAGGGTATCTTGAGCGATGTAGAGCTTCTCAAAGAGATCACTGATGCCTCACGTGGCGCTGTTTCCGCCTTTGTGGAAAAAACAACAAATAGCAAA
GGACAGGTGGTGGATGTGTCTGACAAGCTTACCTCGCTTCTTGGGTTTGGAATCAATGAGCCATGGGTTGAGTATTTGTCCAACACCAAGTTCTACAGGG
CGAACAGAGATAAGCTCAGAACAGTGTTTGGTTTCCTTGGAGAGTGCCTGAAGTTGGTGGTCATGGACAACGAACTAGGGAGTCTAATGCAAGCTTTGGA
AGGCAAGTACGTCGAGCCTGGCCCCGGAGGTGATCCCATCAGAAACCCAAAGGTCTTACCAACCGGTAAAAACATCCATGCCTTAGATCCTCAGGCTATT
CCCACAACAGCAGCAATGGCAAGTGCCAAGATTGTGGTTGAGAGGTTGGTAGAGAGACAGAAGCTCGAAAACGAAGGGAAATATCCCGAGACAATCGCGC
TTGTTCTTTGGGGAACTGACAACATCAAAACATATGGGGAGTCTCTTGGGCAGGTTCTTTGGATGATTGGTGTGAGACCAATTGCTGATACTTTTGGAAG
AGTGAACCGTGTCGAGCCTGTGAGCTTAGAAGAACTAGGAAGGCCGAGGATCGA TGTAGTTGTTAACTGCTCAGGGGTCTTCCGTGATCTCTTTATCAAC
CAGgtaaccagataaaagctttctctcttctttcatttagattcttgttggttggtctctcatggtgtttcttccttgaattatcttgacag ATGAACCT
TCTTGACCGAGCTATCAAGATGGTGGCGGAGCTAGATGAGCCTGTAGAGCAAAATTTTGTAAGGAAACACGCGTTGGAACAAGCAGAGGCGCTTGGCATT
GATATTAGAGAGGCAGCGACAAGAGTTTTCTCAAACGCTTCAGGGTCATACTCAGCCAACATCAGTCTTGCTGTTGAAAACTCGTCATGGAACGATGAGA
AACAGCTTCAGGACATGTACTTGAGCCGCAAATCGTTTGCGTTTGATAGTGATGCTCCTGGAGCAGGAATGGCTGAGAAGAAGCAGGTCTTTGAGATGGC
TCTTAGCACTGCAGAAGTCACCTTCCAGAACCTGGATTCTTCAGAGATTTCTTT GACTGATGTGAGCCACTACTTCGATTCTGACCCTACAAATCTAGTT
CAGAGTTTGAGGAAGGATAAGAAGAAACCAAGCTCTTACATTGCTGACACTACAACTGCAAACGCGCAG gtgagtgaatttctttgtagttcatatatg
Ggatcagttttaccttgcaagaacacattccttaaggtatttgttgaatctggcag GTGAGGACACTATCTGAGACAGTGAGGCTGGACGCAAGAACAAA
GCTGCTGAATCCAAAGTGGTACGAAGGAATGATGTCAAGTGGATATGAAGGAGTTCGTGAGATAGAGAAGAGACTGTCCAACACTGTGGGATGGAGTGCA
ACGTCAGGTCAAGTAGACAATTGGGTCTACGAGGAGGCCAACTCAACTTTCATCCAAGACGAGGAGATGCTGAACCGTCTCATGAACACCAATCCCAACT
CCTTCAGGAAAATGCTTCAGACTTTCTTGGAGGCCAATGGTCGTGGCTACTGGGACACTTC CGCTGAAAACATAGAGAAGCTCAAGGAATTGTACTCGCA
GGTGGAAGACAAGATCGAAGGGATCGATCGATAA

ATG: transcription start site
TGA, TAA: transctiption stop site
Exon
Intron
G-box: CACGTG
Gene: At3g47470, *LHCA4*

ttccgaggttttcaaatcattcaactataataataaatgtttttatttgaatcaaaaactgatcatgggtttgaaactgaactaccacgaagagtctca
atatttctacatatcaaatattccaaagggtacttgggtcggtgaaagcaacaagataataaatttaaaggaaaacgttacaaggaaattgcatggaaat
ttccatgaattggaatccaagacaatgaaagatgctctcactcaattttcaact ctattgtcttgtgttcttggatatgatgagaagttgattacattta
ttattcaactatcaaaatatatataaacatttgtttttttcttatatgacaagattaacttccatgtctccatttggttcagtccaaaaatttcaatttg
attttgagatatatatatatatatatatatatatatatatatatatatatatatactccacctattacttgaaaagccaaacattttcaagaacatat
attaattttgaaatgaaaatacagattaattattaataacaagcattaagtttttattagtatgatcatcattaatgaaagcttgagtcgatggtagttt
cttaggttttctcggtaggtataaaagtatgaatataatactttcaattgaattgcggtgagttttaaagtagcattttctttcctttggtaagtttct
caacaatttacatggagactctttccgcagactttgtgagtaagatatgttttggtcttcg tatacttaactaggaattataaaataggaccaactcaac
acacttattatccaacctttgaaaccaaagaagacaaataaaagcttcatagaattttgtttatggtattccacacacataaatttagatcaatATGGA
ggattcgtctcttcttagcccaatctctctcctcaccattgtcatcttcttattcattctcaatctcatgatgataattcaagacttctcttcttctttt
ccctttcgtttccatctcttcttctctaatgcctacattctcttcacctcaataagaaacaataagcaaaataccgagctcccaatcataaagaaagttg
ttgtcccaaatcgagcagatatcaaaacgtccgtggaagaagtcaaggcgataatcgatgactcggaagctttatacgaatgtttgattgaagaaggaga
agagtacttgcttgagaagaatgagatgatgggtaaggagatagttaaagaggcgtttaggttatttgatgagaatcaggatgggtttatagatgaaaat
gagttaaaacatgtgttgagtttgttaggatacgatgagtgtacaaagatggagtgtaggaagatggttaaggtttatgatgagaatagagatggaaaaa
ttgatttttatgagtttgtgaagcttatagagaagagcttttcgtgaagtagattggaataataataatagttatgttgttggctaattatacgacttgt
ctaatatatttcttcttctttagtgggtcgtcctctaatgaactttattttcctctgacaaacttatttacgttgatatattcatgcttctctgaatttc
caagaaagtgataatatatgacaacgtaaacttaaatttgtgatgaattttgcatggtgatttccagttaggtttggatatcgtctttatctataatatt
gaagggaaaaaagtatggaccataatcgaacaaataaacttaattttcaaaaaactacagttaaaaatagtttttttgaattcacaagttctctctagtag
actttgataagagttttaatggatagaatttgtatttactttcacacttataataataaaagtgatcatacataatgtttatatagagcttgagactgc
tacatctagctagtttcctaatacacttttcaataataaaaagagaagagatt aatttgttttttttttaaaaaggtcaagataatagtcaatctcatc
atttgctccaaaatttagtgttaatcaaatgtgggattatataacagactacaatgtaaatagtaggattctacaaactcaaaaatatttgtaacagtt
ttttcttttaaaatggctttcaaattttgaggccgaatcgatggcttatgtatcttgttttctggggtctggctagatcatctgttagcacactaaattt
tgtaatttcctacaaaatgcatgaagaatattcccaaaatatcttataaggatcttcagtatcttctaatccattataagatttgagttaacagtttaaa
aggtaatctcaaatgtcactgtaactctgtaagattttgaaagatgaacatgatcttgaaaaagagagattaaagaaaaagatggatttaagttcttcaa
cactctctttcttcttttcttcttgggcctaaaacaacccaatatctcactcttcaaacga gcccgagattctcaaccttgacaagcccaaagtgaagat
agtttttagataaatccaaattggttttatgttttacaagttacatacactcccctatcttgatgattaggaaatcagactttagattttggtttcc
catatgccaattcagttccatttgattggttgacgagaatcctcactccactctaatatttttaaatccatttaattga cacgtgtaacaacaatggacc

ctcctccgtcaaatcaaccacgtcatctaatccaacggtagatactcagtcatcaaaccacagccacaaaactcgtttcaagaaacgaataaacttacaa
aatcccctccaagtcttatttcttcacaaccatctctgaactcatctttcaatctctcttgattccaatccattcttcttcaagtgccgggttctcgggt
cggatcttctaagtctagctagggataATGGCTACTGTCACTACTCATGCCTCGGCCTCGATTTTCCGACCTTGTACTTCAAAGCCAAGATTCCTCACCG
GTTCTTCCGGTAGGTTGAACCGCGACTTGTCGTTTACATCGATCGGTTCATCGGCCAAAACGTCGTCGTTCAAGGTTGAAGCTAAG
AAAGGAGAATGGTTGCCCGGTTTGGCATCGCCTGATTATCTCACCGGCAGgtaatgttttgcaaactacattcaatttcggtttaa
cacaaccggaaccaacatggtaatcgatttggttagcttaggacagtccgataccggtttagtttaagtgtataggaatttgtttg
aacacaaccgtgactaatcagtttggattggttaggttagaccggacttggttgataccggtttagttaataatggtcactcactc
actcttatacatattgcagTCTTGCCGGTGACAATGGGTTTGACCCGTTGGGTCTAGCAGAGGATCCAGAGAACTTGAAATGGTTC
GTCCAGGCAGAGCTGGTCAACGGACGATGGGCTATGCTCGGTGTCGCTGGGATGCTTTTGCCGGAAGTTTTCACCAAGATCGgtga
gttaccattaacatgaccttaaaacaatttataacttctttcaagaacttaacacattgtcaaaactgttggtagGAATCATAAAT
GTTCCTGAGTGGTACGATGCTGGGAAAGAGCAGTATTTTGCATCGTCGTCGACATTGTTCGTGATCGAGTTCATATTGTTTCATTA
CGTTGAGATCAGACGGTGGCAAGACATCAAGAACCCAGGAAGTGTGAACCAAGACCCTATCTTTAAGCAATACAGCTTACCTAAGG
GTGAAGTTGGTTACCCTGGTGGAATCTTTAACCCGCTTAACTTTGCTCCTACGCAAGAGGCCAAGGAGAAAGAGCTAGCAAACGgt
aagcttagactaatccgctattcttgttatgcaagaaactgctagttgtagttaaaagacttaaaagacatttttgttgttgttgc
agGGAGGTTGGCGATGTTGGCATTCTTAGGGTTTGTGGTTCAACACAATGTGACTGGAAAAGGACCATTTGAGAATCTGTTGCAGC
ACTTGTCTGACCCATGGCACAACACTATTGTCCAAACCTTCAACTAA

Figure S2
