# Supplementary material for: The HY5-PIF Regulatory Module Coordinates Light and Temperature Control of Photosynthetic Gene Transcription
Source: PLoS Genet. 2014 Jun 12;10(6):e1004416. doi: 10.1371/journal.pgen.1004416 (PMC4055456; doi:10.1371/journal.pgen.1004416)
Supplement: Table S1 — List of primers used for qPCR analyses, EMSA assays and ChIP tests. (DOCX) [file pgen.1004416.s010.docx]

**Supplementary Table 1. Oligonucleotides used in this work.**

| qVDE F | GCGTTCCTTATTGTTCCATCTG | | | |  | |  | |
| --- | --- | --- | --- | --- | --- | --- | --- | --- |
| qVDE R | CCCACATTTAATCTGGCACTC | | | |  | |  | |
|  |  |  |  |  | |  | |  |
| qGUN5 F | CTGGTCGTGACCCTAGAACAG | | | |  | |  | |
| qGUN5 R | GATTGCCAGCTTCTTCTCTG | | | |  | |  | |
|  |  |  |  |  | |  | |  |
| qPorC F | GGGCAAAACAGTTCAATGA | | | |  | |  | |
| qPorC R | GGAAAAAGAAGCCGAAACAG | | | |  | |  | |
|  |  |  |  |  | |  | |  |
| qLHCA4 F | GCATCGCCTGATTATCTCAC | | | |  | |  | |
| qLHCA4 R | AACATTTATGATTCCGATCTTGGT | | | |  | |  | |
|  |  |  |  |  | |  | |  |
| qPSY F | GACACCCGAAAGGCGAAAGG | | | |  | |  | |
| qPSY R | CAGCGAGAGCAGCATCAAGC | | | |  | |  | |
|  |  |  |  |  | |  | |  |
| qACT7 F | CAGTGTCTGGATCGGAGGAT | | | |  | |  | |
| qACT7 R | TGAACAATCGATGGACCTGA | | | |  | |  | |
| qHY5 F | AAACAGAGTGAAAGACTTGGAG |  |  |  | |  | |  |
| qHY5 R | CTTCAGAATATGTCTAAGCATCTGG | | | |  | |  | |
| **Oligos for ChIP** |  |  |  |  | |  | |  |
| qGUN5(G+) F | ACTCAACCTCACAAGTAGACTC | | | |  | |  | |
| qGUN5(G+) R | TAAGTGGTGAGAGGGAAGGA | | | |  | |  | |
|  |  |  |  |  | |  | |  |
| qPorC(G+) F | GGGCAAAACAGTTCAATGA | | | |  | |  | |
| qPorC(G+) R | GGAAAAAGAAGCCGAAACAG | | | |  | |  | |
|  |  |  |  |  | |  | |  |
| qLHCA4(G+) F | TGGTTGACGAGAATCCTCAC | | | |  | |  | |
| qLHCA4(G+) R | GAATAAGATAGTGTCGGGTCCA | | | |  | |  | |
|  |  |  |  |  | |  | |  |
| qPSY(G+) F | TGGGACCCAAATACCGACTA | | | |  | |  | |
| qPSY(G+) R | CGAGGGTTGCGTAGAAAACT | | | |  | |  | |
|  |  |  |  |  | |  | |  |
| **EMSA** |  |  |  |  | |  | |  |
| G-box I F | CCATTGGCCGTAGGCTC**CACGTG**GCATCTCTCTGATCT | | | | | | | |
| G-box I R | AGATCAGAGAGATGC**CACGTG**GAGCCTACGGCGAATGG | | | | | | | |
|  |  |  |  |  | |  | |  |
| G-mut I F | CCATTGGCCGTAGGCTCCTTTTTTGCATCTCTCTGATCT | | | | | | | |
| G-mut I R | AGATCAGAGAGATGCCAAAAAGAGCCTACGGCCAATGG | | | | | | | |
